# Mycotoxins in poultry feed and feed ingredients in Nigeria

**DOI:** 10.1007/s12550-018-0337-y

**Published:** 2018-11-27

**Authors:** Oyekemi O. Akinmusire, Abdul-Dahiru El-Yuguda, Jasini A. Musa, Oluwawapelumi A. Oyedele, Michael Sulyok, Yinka M. Somorin, Chibundu N. Ezekiel, Rudolf Krska

**Affiliations:** 1 Department of Microbiology, University of Maiduguri, Maiduguri, Borno Nigeria; 2 Department of Veterinary Microbiology, University of Maiduguri, Maiduguri, Borno Nigeria; 3 Department of Microbiology, Babcock University, Ilishan Remo, Ogun Nigeria; 4 Center for Analytical Chemistry, Department of Agrobiotechnology (IFA-Tulln), University of Natural Resources and Life Sciences Vienna (BOKU), Konrad-Lorenz-Str. 20, 3430 Tulln, Austria; 5 Microbiology, School of Natural Sciences, National University of Ireland, Galway, Ireland; 6 Institute for Global Food Security, School of Biological Sciences, Queen’s University Belfast, University Road, Belfast, BT7 1NN UK

**Keywords:** Aflatoxin, Fumonisin, Peanut, Maize, Mycotoxins, Poultry

## Abstract

Mycotoxins are toxic secondary fungal metabolites that can negatively affect animal productivity when ingested through feed. In order to assess mycotoxin contamination of poultry feed and feed ingredients vis-a-vis source tracking of feed contamination in Nigeria, 102 samples of feed (*n* = 30) and feed ingredients (*n* = 72) were collected from in-house mills of poultry farms across 12 states of Nigeria and analyzed for multiple mycotoxins using LC/MS-MS. One hundred and forty microbial metabolites were detected in the feed and feed ingredients. The most frequent mycotoxin in the feed was fumonisin B_1_, occurring in 97% of the samples at mean concentration of 1014 μg kg^−1^. AFB_1_ occurred in 83% of the feed samples at mean concentration of 74 μg kg^−1^ and in all feed ingredients except fish meal and other cereals (millet and rice). Feed samples analyzed in this study were contaminated with at least four mycotoxins: aflatoxins and fumonisin co-occurring in 80% of the samples. Peanut cake and maize contributed the most to the levels of aflatoxin and fumonisin, respectively, in the feed. Consequently, there is a need to explore other cereal- and protein-based ingredients for compounding feeds in order to reduce the risk associated with high mycotoxin (e.g. aflatoxin) intake in poultry.

**Electronic supplementary material:**

The online version of this article (10.1007/s12550-018-0337-y) contains supplementary material, which is available to authorized users.

## Introduction

The poultry industry in Nigeria is an essential subsector of agriculture that provides food, employment, and other economic resources for the country (Ezekiel et al. 2012a). Livestock production can be threatened when feeds are contaminated by fungi and their toxic metabolites. Several mycotoxins, including aflatoxins (AFs), cyclopiazonic acid (CPA), fumonisins (FUMs), nivalenol (NIV) and zearalenone (ZEN) have been reported to contaminate poultry feed and their ingredients (Labuda et al. 2005; De Boevre et al. 2012; Ezekiel et al. 2012a; Njobeh et al. 2012; Rodrigues and Naehrer 2012; Abia et al. 2013a; Kana et al. 2013; Streit et al. 2013a, 2013b). The occurrence of mycotoxins in feed ingredients depends on several factors that include climatic conditions, diversity of fungi contaminating the crops, harvesting methods of the individual crops, storage practices, and seasonal variations, while the types and levels of mycotoxins in the feed largely depend on the mycotoxins in the individual feed ingredients, the mix/proportion of feed ingredients, feed processing techniques, and storage practices (Warth et al. 2012; Ezekiel et al. 2014).

Mycotoxins pose a huge threat to the safety and security of livestock first and then to human beings that consume them due to their different toxic effects and their probable synergistic properties (Shephard 2008; Hossain et al. 2011; Njobeh et al. 2012). When animals ingest feed contaminated with high mycotoxin concentrations, mycotoxicoses, often marked by reduced animal productivity (reduced body weight gain, reduced litter sizes, deformed offspring, reduced egg production) and immune suppression (Shareef 2010; Hossain et al. 2011; Streit et al. 2013a), could result to severe economic losses.

Poultry feed ingredients are derived from a variety of raw materials that originate from plants and animals. It is usually a mixture of cereals (mostly maize) that serves as energy source, animal protein sources (fish meal, meat, and bone meal), and plant protein sources (soybean meal and peanut). Maize, the predominant grain used in poultry feeds, can be contaminated by mycotoxins from *Aspergillus*, *Fusarium* and *Penicillium* during processing and storage (Zinedine and Manes 2009; Adetunji et al. 2014). Peanut and its processed products, peanut cake, have been found to be highly susceptible to aflatoxin contamination (Ezekiel et al. 2012b, 2013; Kayode et al. 2013; Oyedele et al. 2017; Ginting et al. 2018).

Globally, mycotoxins in finished poultry feed have been reported (Labuda et al. 2005; De Boevre et al. 2012; Ezekiel et al. 2012a; Njobeh et al. 2012; Rodrigues and Naehrer 2012; Abia et al. 2013a; Kana et al. 2013; Streit et al. 2013b), but there is sparse information on the source tracking of mycotoxin contamination of the feed by individual ingredients, especially in Nigeria. Hence, this study aimed at investigating mycotoxins in poultry feed and the ingredients used in locally formulating the feed in Nigeria with a view to associate contamination of major ingredients to overall contamination in finished feed. This mini-survey provides snapshot data for the consideration of other cereal-based ingredients and protein sources that are less prone to AFs and FUMs contamination in feed formulation.

## Materials and methods

### Sampling plan and collection of feed samples

Poultry feed and feed ingredients were collected from feed mills in 12 states of Nigeria. The states were primarily selected based on accessibility for sampling and they include Adamawa, Benue, Borno, Delta, Kaduna, Katsina, Kebbi, Lagos, Niger, Oyo, Rivers, and Taraba states. Only in-house feed mills (i.e., feed mills owned by and situated at poultry farms) that had at least 40 bags each of feed and feed ingredients at the time of sampling were considered in the study. The poultry farms were thus the largest farms with in-house mills in the study states. Consequently, only one feed mill was selected per state and at least two feed samples were collected per feed mill. The poultry feed samples included growers’ mash, finisher feed, and layers’ mash, and the collection depended on poultry farm specialization. For every two feed samples collected, one set of ingredients (comprising of individual ingredients depending on use per mill) was obtained. Both feed (*n* = 30) and feed ingredients (*n* = 72) were sampled from bulk (50 kg) bags, and the feed ingredients collected were those used in the formulation of the feeds that were sampled. Feed ingredients were from local produce, and they include maize (*n* = 17), peanut cake (*n* = 11), wheat offal (*n* = 10), other cereals (*n* = 6), soybean (*n* = 11), bone (*n* = 9), fish meal (*n* = 5), and palm kernel (*n* = 3). Samples (*n* = 102) were collected for this study during June 2013.

Each sample (4 kg) consisted of four 1 kg representative subsamples: each pooled from a bulk bag of feed/ingredient that was randomly selected out of at least ten feed/ingredient bags. The four randomly selected bags for sampling of feed/ingredient were from the same batch of feed/ingredient in order to reduce variability and ensure the batch was well represented. Each 1 kg subsample consisted of three portions of respective feed/ingredient that weighed 300–350 g. The subsamples were collected by manually probing three points (top, middle, and bottom) of the 50 kg feed/ingredient bags. The samples were thoroughly mixed and quartered successively to give representative samples applied to multi-microbial metabolite analysis.

### Quantification of microbial metabolites in feed and feed ingredients

#### Chemicals

Methanol (LC gradient grade) and glacial acetic acid (p.a.) were purchased from Merck (Darmstadt, Germany), acetonitrile (LC gradient grade) from VWR (Leuven, Belgium), and ammonium acetate (MS grade) from Sigma-Aldrich (Vienna, Austria). Standards for fungal and bacterial metabolites were obtained from various research groups and/or commercial sources. Water was purified successively by reverse osmosis with an Elga Purelab ultra analytic system from Veolia Water (Bucks, UK).

#### Extraction and estimation of matrix effects

Five grams of each representative sample were weighed into a 50-ml polypropylene tube (Sarstedt, Nümbrecht, Germany) and 20 ml of the extraction solvent (acetonitrile/water/acetic acid 79:20:1, *v*/*v*/*v*) added. For spiking experiments, 0.25 g samples were used for extraction. Samples were extracted for 90 min on a GFL 3017 rotary shaker (GFL, Burgwedel, Germany) and diluted with the same volume of dilution solvent (acetonitrile/water/acetic acid 20:79:1, *v*/*v*/*v*), and the diluted extracts were injected into the LC instrument (Sulyok et al. 2006). Centrifugation was not necessary due to sufficient sedimentation by gravity. Apparent recoveries of the analytes were cross-checked by spiking three different samples that were not contaminated with mycotoxins with a multi-analyte standard on one concentration level, since previously generated data are available (Ezekiel et al. 2012a; Warth et al. 2012; Abia et al. 2013b).

#### LC-MS/MS parameters

LC-MS/MS screening of target microbial metabolites was performed with a QTrap 5500 LC-MS/MS System (Applied Biosystems, Foster City, CA, USA) equipped with TurboIonSpray electrospray ionization (ESI) source and a 1290 Series HPLC System (Agilent, Waldbronn, Germany). Chromatographic separation was performed at 25 °C on a Gemini® C18-column, 150 × 4.6 mm ID, 5 μm particle size, equipped with a C18 4 × 3 mm ID security guard cartridge (Phenomenex, Torrance, CA, USA). The chromatographic method, chromatographic, and mass spectrometric parameters are as described by Malachová et al. (2014). ESI-MS/MS was performed in the time-scheduled multiple reaction monitoring (MRM) mode both in positive and negative polarities in two separate chromatographic runs per sample by scanning two fragmentation reactions per analyte. The MRM detection window of each analyte was set to its expected retention time ± 27 and ± 48 s in the positive and the negative modes, respectively. Confirmation of positive analyte identification was obtained by the acquisition of two MRMs per analyte (with the exception of moniliformin (MON), which exhibited only one fragment ion). This yielded 4.0 identification points according to European Commission decision 2002/657 (EU 2002). In addition, the LC retention time and the intensity ratio of the two MRM transitions agreed with the related values of an authentic standard within 0.1 min and 30%, respectively. The accuracy of the method is monitored on a routine basis by regular participation in proficiency testing organized by BIPEA (Gennevilliers, France). Eight hundred and twenty-four out of 875 results submitted overall, and 121 out of 129 results submitted for animal feed were in the satisfactory range of − 2 < *z* < 2 (results until March 2018 included).

## Results and discussion

### Overview of multiple microbial metabolite occurrences in feed and ingredients

The performance of the LC-MS/MS method is described in Table S1. A total of 140 microbial metabolites were detected in the feed (121 metabolites; Tables 1 and S2) and feed ingredients (132 metabolites; Tables 2, 3, S3, and S4). Major mycotoxins such as AFs, DON, FUMs, NIV, ochratoxin A (OTA), T-2 and HT-2, ZEN and their metabolites were found to contaminate the compounded feed and feed ingredients at different incidences and concentrations. The spectrum of metabolites including mycotoxins reported herein are quite similar to the metabolite diversity in Streit et al. (2013b) but more than those previously reported in feed and feed ingredients (De Boevre et al. 2012; Ezekiel et al. 2012a; Njobeh et al. 2012; Rodrigues and Naehrer 2012; Abia et al. 2013a; Streit et al. 2013b).

**Table 1:** Occurrence levels of 23 major mycotoxins in 30 poultry feed samples from Nigeria

Metabolites	Percent^a^	Concentration (μg/kg)		
		Min	Max	Mean
Aflatoxin B_1_	83.3	0.5	760	74
Aflatoxin B_2_	50.0	1.7	188	21
Aflatoxin G_1_	56.7	1.6	79	19
Aflatoxin G_2_	13.3	0.5	7.6	3.5
Aflatoxin M_1_	23.3	1.7	41	9.9
Alternariol (AOH)	40.0	0.2	8.6	2.7
AOHmethylether	46.7	0.2	5.6	1.4
Beauvericin	100	0.5	127	13
Citrinin	16.7	38	2340	522
Cyclopiazonic acid	10.0	23	49	39
Deoxynivalenol	20.0	36	174	108
Fumonisin B_1_ (FB_1_)	96.7	37	3760	1014
Fumonisin B_2_	93.3	9.2	870	310
Fumonisin B_3_	90.0	9.0	149	62
Fumonisin B_4_	96.7	3.3	168	623
Hydrolyzed FB_1_	56.7	5820	86,800	28,958
Moniliformin	93.3	5.1	900	62
Nivalenol	23.3	13	647	114
Ochratoxin A	26.7	0.8	15	5.4
Ochratoxin B	20.0	1.2	24	9.3
Tenuazonic acid	70.0	5.2	315	44
Zearalenone (ZEN)	83.3	0.5	71	9.3
ZEN-sulfate	13.3	3.2	162	56

^a^Incidence of contamination expressed in percentage

**Table 2:** Distribution of 23 major mycotoxins in 44 cereal and nut ingredients for poultry feed in Nigeria

Metabolites	Maize (*n*^a^ = 17; *n*^b^ = 84)				Peanut cake (*n*^a^ = 11; *n*^b^ = 68)				Wheat offal (*n*^a^ = 10; *n*^b^ = 106)				Other cereals (*n*^a^ = 6; *n*^b^ = 39)			
	Percent^c^	Concentration (μg/kg)			Percent^c^	Concentration (μg/kg)			Percent	Concentration (μg/kg)			Percent^c^	Concentration (μg/kg)		
		Min	Max	Mean		Min	Max	Mean		Min	Max	Mean		Min	Max	Mean
Aflatoxin B_1_	47.1	6.1	567	176	90.9	61	3860	639	30.0	1.3	80	53	n.d	n.d	n.d	n.d
Aflatoxin B_2_	23.5	3.3	61	35	90.9	6.6	895	126	10.0	5.9	5.9	0.0	n.d	n.d	n.d	n.d
Aflatoxin G_1_	41.2	2.0	725	110	90.9	17	568	157	20.0	13	14	14	n.d	n.d	n.d	n.d
Aflatoxin G_2_	5.9	60	60	0.0	54.5	2.5	68	27	n.d	n.d	n.d	n.d	n.d	n.d	n.d	n.d
Aflatoxin M_1_	17.6	25	70	45	72.7	14	254	49	20.0	5.1	5.3	5.2	16.7	1.6	1.6	0.0
Alternariol (AOH)	11.8	0.4	0.4	0.4	n.d	n.d	n.d	n.d	70.0	2.7	23	12	n.d	n.d	n.d	n.d
AOHmethylether	23.5	0.3	1.0	0.5	9.1	0.1	0.1	0.0	90.0	0.4	8.9	4.2	n.d	n.d	n.d	n.d
Beauvericin	100	0.1	33	7.7	100	0.8	9.7	2.3	90.0	2.3	37	13	83.3	1.4	8.7	5.6
Citrinin	17.6	789	9400	4229	9.1	150	150	0.0	n.d	n.d	n.d	n.d	n.d	n.d	n.d	n.d
Cyclopiazonic acid	5.9	98	98	0.0	27.3	34	204	93	n.d	n.d	n.d	n.d	n.d	n.d	n.d	n.d
Deoxynivalenol	n.d	n.d	n.d	n.d	n.d	n.d	n.d	n.d	50.0	348	837	578	16.7	22	22	0.0
Fumonisin B_1_ (FB_1_)	100	164	2090	825	27.3	4.7	910	308	50.0	2.8	67	37	16.7	0.9	0.9	0.0
Fumonisin B_2_	100	46	710	262	18.2	0.9	340	171	50.0	1.3	15	7.9	16.7	1.5	1.5	0.0
Fumonisin B_3_	100	10	186	69	9.1	62	62	0.0	10.0	6.9	6.9	0.0	n.d	n.d	n.d	n.d
Fumonisin B_4_	100	16	253	98	9.1	55	55	0.0	40.0	3.5	6.0	5.0	n.d	n.d	n.d	n.d
Hydrolyzed FB_1_	76.5	3500	80,500	24,089	n.d	n.d	n.d	n.d	10.0	3150	3150	0.0	n.d	n.d	n.d	n.d
Moniliformin	88.2	12	246	74	81.8	0.3	16	6.0	100	4.8	60	17	66.7	4.5	307	102
Nivalenol	23.5	9.7	17	14	9.1	64	64	0.0	10.0	4.7	4.7	0.0	n.d	n.d	n.d	n.d
Ochratoxin A	11.8	1.3	3.1	2.2	54.5	0.1	127	35	20.0	0.6	1.0	0.8	33.3	0.5	3.6	2.0
Ochratoxin B	5.9	3.7	3.7	0.0	18.2	158	302	230	n.d	n.d	n.d	n.d	n.d	n.d	n.d	n.d
Tenuazonic acid	5.9	7.8	7.8	0.0	n.d	n.d	n.d	n.d	90.0	60	679	190	66.7	34	80	53
Zearalenone (ZEN)	64.7	0.1	4.8	1.2	18.2	0.7	1.1	0.9	90.0	0.4	67	19	n.d	n.d	n.d	n.d
ZEN-sulfate	n.d	n.d	n.d	n.d	n.d	n.d	n.d	n.d	20.0	31.8	33	33	n.d	n.d	n.d	n.d

*n.d* not detected

^a^Number of samples analyzed

^b^Number of metabolites detected

^c^Incidence of contamination expressed in percentage

**Table 3:** Occurrence levels of 23 major mycotoxins in 28 other ingredients for poultry feed in Nigeria

Metabolites	Bone (*n*^a^ = 9; *n*^b^ = 59)				Fish meal (*n*^a^ = 5; *n*^b^ = 25)				Palm kernel (*n*^a^ = 3; *n*^b^ = 68)				Soybean (*n*^a^ = 11; *n*^b^ = 52)			
	Percent^c^	Concentration (μg/kg)			Percent^c^	Concentration (μg/kg)			Percent^c^	Concentration (μg/kg)			Percent^c^	Concentration (μg/kg)		
		Min	Max	Mean		Min	Max	Mean		Min	Max	Mean		Min	Max	Mean
Aflatoxin B_1_	33.3	1.9	52	19	n.d	n.d	n.d	n.d	100	32	397	162	45.5	0.7	91	38
Aflatoxin B_2_	11.1	9.0	9.0	0.0	n.d	n.d	n.d	n.d	100	6.9	57	25	18.2	5.9	8.0	6.9
Aflatoxin G_1_	11.1	6.3	6.3	0.0	n.d	n.d	n.d	n.d	100	6.5	198	71	45.5	0.3	20	4.9
Aflatoxin G_2_	n.d	n.d	n.d	n.d	n.d	n.d	n.d	n.d	33.3	12	12	0.0	n.d	n.d	n.d	n.d
Aflatoxin M_1_	n.d	n.d	n.d	n.d	n.d	n.d	n.d	n.d	33.3	21	21	0.0	9.1	3.5	3.5	0.0
Alternariol (AOH)	n.d	n.d	n.d	n.d	n.d	n.d	n.d	n.d	100	4.0	12	8.1	9.1	1.3	1.3	0.0
AOHmethylether	11.1	0.6	0.6	0.0	n.d	n.d	n.d	n.d	100	7.1	8.8	8.1	18.2	0.2	0.6	0.4
Beauvericin	11.1	4.2	4.2	0.0	100	0.1	0.5	0.3	100	0.2	9.9	3.5	90.9	0.5	4.8	1.5
Cyclopiazonic acid	n.d	n.d	n.d	n.d	n.d	n.d	n.d	n.d	33.3	44	44	0.0	n.d	n.d	n.d	n.d
Fumonisin B_1_ (FB_1_)	n.d	n.d	n.d	n.d	n.d	n.d	n.d	n.d	66.7	58	122	90	18.2	44	46	45
Fumonisin B_2_	n.d	n.d	n.d	n.d	n.d	n.d	n.d	n.d	66.7	29	33	31	18.2	12	17	15
Fumonisin B_3_	n.d	n.d	n.d	n.d	n.d	n.d	n.d	n.d	33.3	4.1	4.1	0.0	9.1	1.8	1.8	0.0
Fumonisin B_4_	11.1	0.9	0.9	0.0	n.d	n.d	n.d	n.d	66.7	5.2	8.4	6.8	9.1	5.6	5.6	0.0
Hydrolyzed FB_1_	n.d	n.d	n.d	n.d	n.d	n.d	n.d	n.d	33.3	5750	5750	0.0	n.d	n.d	n.d	n.d
Moniliformin	n.d	n.d	n.d	n.d	n.d	n.d	n.d	n.d	100	7.2	17	14	18.2	28	63	45
Ochratoxin A	11.1	0.1	0.1	0.0	n.d	n.d	n.d	n.d	33.3	1.0	1.0	0.0	9.1	3.7	3.7	0.0
Ochratoxin B	n.d	n.d	n.d	n.d	n.d	n.d	n.d	n.d	n.d	n.d	n.d	n.d	9.1	15	15	0.0
Tenuazonic acid	11.1	22	22	0.0	100	13	47	36	33.3	4.2	4.2	0.0	27.3	12	128	55
Zearalenone (ZEN)	11.1	6.5	6.5	0.0	n.d	n.d	n.d	n.d	66.7	0.3	0.6	0.4	54.5	0.3	1.0	0.6
ZEN-sulfate	11.1	1.2	1.2	0.0	n.d	n.d	n.d	n.d	n.d	n.d	n.d	n.d	n.d	n.d	n.d	n.d

*n.d* not detected

^a^Number of samples analyzed

^b^Number of metabolites detected

^c^Incidence of contamination expressed in percentage

### Occurrence of major mycotoxins in feed and feed ingredients

Twenty-three mycotoxins were found in the compounded feed samples (Table 1) and their ingredients (Tables 2 and 3). The most frequently detected mycotoxin in the feed was fumonisin B_1_ (FB_1_; incidence, 97%; range, 37–3760 μg kg^−1^; mean, 1014 μg kg^−1^). FUMs were also quantified in all feed ingredients except bone and fish meal (Tables 2 and 3). Fumonisin B_1_ occurred in all (100%) the maize samples (range, 164–2090 μg kg^−1^; mean, 825 μg kg^−1^), and surprisingly was detected in 27% of 11 peanut cake samples due to co-storage of bags of maize grains and processed peanut cake in non-ventilated warehouses leading to deposited maize grain dust on the peanut cakes. The reported incidence and concentration of FUMs in feed samples in the present study are higher than previous reports of relatively high FUM levels in feed from poultry farms in Cameroon (100%; range, 16–1930 μg kg^−1^; mean, 468 μg kg^−1^; Abia et al. 2013a), commercially produced feed from Nigeria (83%; range, 31–2733 μg kg^−1^; mean, 964 μg kg^−1^; Ezekiel et al. 2012a), and commercially compounded feed from South Africa (87%; range, 104–2999 μg kg^−1^; mean, 903 μg kg^−1^; Njobeh et al. 2012). The high-FUM contamination level of feed in the present study may reflect the higher FUM contamination of maize samples used to formulate the feed samples in our study compared to the commercially processed feed analyzed by the other studies. This is also suggested by the significant correlation (*r*^2^ = 0.405, *p* = 0.03) obtained for total FUM concentrations in maize and in the compounded feed.

Aflatoxin B_1_ was detected in 83% of the analyzed feed samples (range, 0.5–760 μg kg^−1^; mean, 74 μg kg^−1^) (Table 1) and in all feed ingredients except fish meal and other cereals (millet and rice; Tables 2 and 3). The AFB_1_ content in feed ingredients reached 397 μg kg^−1^ and 3860 μg kg^−1^ in palm kernel and peanut cake, respectively, with the highest mean level (639 μg kg^−1^) recorded in peanut cake and the lowest found in bone (19 μg kg^−1^). Other aflatoxin types, B_2_, G_1_, G_2_, and M_1_, were also found in the feed and ingredient samples albeit at lower incidences (and levels). The vast contamination of the feed and feed ingredients with AFs agrees with several previous reports of high aflatoxin contamination of cereals, nuts, legumes, oilseeds, and their products in Nigeria (Ezekiel et al. 2012b, 2013; Adetunji et al. 2014; Egbontan et al. 2017; Oyedele et al. 2017) as well as with the aflatoxin contamination of feed samples from different countries (Cameroon, India, Nigeria, and South Africa), albeit at a relatively higher contamination level than samples from these previous studies (Oluwafemi et al. 2009; Njobeh et al. 2012; Abia et al. 2013a; Kehinde et al. 2014; Kotinagu et al. 2015). However, our previous paper on commercial poultry feed in Nigeria reported higher concentrations (max, 1067 μg kg^−1^; mean, 198 μg kg^−1^) (Ezekiel et al. 2012a) than the present study. The disparity in aflatoxin contamination data in the several studies including the present paper may be attributed to a combination of factors ranging from climatic factors, agricultural, and processing (handling and storage) practices for raw materials, to the formulation mix utilized during compounding of the feed. A significant correlation (*r*^2^ = 0.473, *p* = 0.03) was recorded for AFB_1_ levels in peanut cake and in the analyzed feed. This agrees with the report of Atawodi et al. (1994) that food and feed containing peanut are most contaminated with AFs: a possible reason for the high AF levels in feed samples in the present study.

Other major mycotoxins found in the analyzed feed samples include citrinin (CIT), deoxynivalenol (DON), NIV, OTA, and ZEN (Table 2). Citrinin was detected in 17% of the feed (max, 2340 μg kg^−1^; mean, 522 μg kg^−1^), while the trichothecenes, DON and NIV, contaminated at least 20% of the samples at concentrations reaching 174 μg kg^−1^ (mean, 108 μg kg^−1^) and 647 μg kg^−1^ (mean, 114 μg kg^−1^), respectively. The mean concentrations of OTA and ZEN in the feed were less than 10 μg kg^−1^. The mean levels for the aforementioned mycotoxins, except CIT, as observed in this study, are lower than those previously reported in commercial feed from Nigeria (Ezekiel et al. 2012a) and South Africa (Njobeh et al. 2012), and from feed collected on farms in Cameroon (Abia et al. 2013a). Similar to the report of Abia et al. (2013a), CIT was found in feed samples, albeit at much higher concentrations. Furthermore, we document the uncommon presence of tenuazonic acid in feed and almost all the ingredients; a mycotoxin recently reported in members of the *Aspergillus* section *Flavi* (Frisvad et al. 2019), which are common contaminants of several stored food items including grains and fish products.

Overall, all feed samples in this study were contaminated with at least four mycotoxins, with AFs and FUM co-occurring in 80% of the samples. Mixtures of several mycotoxins such as those observed in this study have been suggested to induce a range of antagonistic, additive, or synergistic effects in various cells and organs of animals including poultry (Grenier and Oswald 2011); however, the toxicity effects of mycotoxin combinations are not always predicted based on individual toxin toxicities. In addition, despite the toxin contamination data shown in this mini-survey, categorical views on the possible adverse health impacts will be premature in view of the low numbers of feed and ingredient samples analyzed and underrepresentation of the country in this study.

This mini-survey has shown that mycotoxin contamination of locally formulated poultry feed in some parts of Nigeria may be high, with maize and peanut contributing significantly to the respective FUM and AF levels in the studied feed samples. The co-contamination of feed samples with diverse mycotoxins/metabolites of varying concentrations suggests possible health risk to the animals and reduced profitability for the farmers. The following options are therefore suggested for implementation in an integrated manner to control mycotoxins in the poultry sector: (1) adoption of good agricultural practices including the application of available biological control products to crops in order to lower the contamination levels, (2) provision of good storage conditions for grains intended for poultry feed formulation to limit fungal proliferation and further toxin accumulation, (3) monitoring of mycotoxins in locally compounded feed and feed ingredients, (4) exploration of alternative and easily accessible crops (e.g., bambara nut, millet, sorghum) that may be less prone to AF and FUM contamination, and (5) educational training programs on mycotoxin reduction strategies for farmers and millers involved with the poultry industry.

## Electronic supplementary material

ESM 1 (DOCX 158 kb)
