# Supplementary material for: Mycotoxins in poultry feed and feed ingredients in Nigeria
Source: Mycotoxin Res. 2018 Nov 27;35(2):149–55. doi: 10.1007/s12550-018-0337-y (PMC6478637; doi:10.1007/s12550-018-0337-y)
Supplement: Supplementary file 1 — (DOCX 158 kb) [file 12550_2018_337_MOESM1_ESM.docx]

Mycotoxins in poultry feed and feed ingredients in Nigeria

Oyekemi O. Akinmusire^a^ , Abdul-Dahiru El-Yuguda^b^, Jasini A. Musa^b^, Oluwawapelumi A. Oyedele^c^, Michael Sulyok^d^, Yinka M. Somorin^e^, Chibundu N. Ezekiel^c,d^* & Rudolf Krska^d,f^

^a^Department of Microbiology, University of Maiduguri, Maiduguri, Borno State, Nigeria. ^b^Department of Veterinary Microbiology, University of Maiduguri, Maiduguri, Borno State, Nigeria. ^c^Department of Microbiology, Babcock University, Ilishan Remo, Ogun State, Nigeria. ^d^Center for Analytical Chemistry, Department of Agrobiotechnology (IFA-Tulln), University of Natural Resources and Life Sciences Vienna (BOKU), Konrad Lorenzstr. 20, A-3430 Tulln, Austria. ^e^Microbiology, School of Natural Sciences, National University of Ireland, Galway, Ireland. ^f^Institute for Global Food Security, School of Biological Sciences, Queen’s University Belfast, University Road, Belfast, BT7 1NN, Northern Ireland, United Kingdom.

*Corresponding author: [chaugez@gmail.com](mailto:chaugez@gmail.com) (C.N. Ezekiel)

**Table S1** LC-MS/MS performance characteristics^a^ for 140 microbial metabolites in poultry feed and feed ingredients.

| Metabolite | Recovery (%) | | | | | |  | Metabolite | Recovery (%) | | | | | |
| --- | --- | --- | --- | --- | --- | --- | --- | --- | --- | --- | --- | --- | --- | --- |
|  | Feed | GNC^b^ | Maize | Rice | Millet | Wheat |  |  | Feed | GNC^b^ | Maize | Rice | Millet | Wheat |
| 15-Hydculmorin | 100 | 77 | 92.8 | 103.7 | 98.8 | 97.5 |  | Bis(methylthio)gliotoxin | 87.7 | 83.5 | 85 | 85 | 85 | 85 |
| 15-Hydroxyculmoron | 100 | 60.9 | 100 | 102 | 97.2 | 100 |  | Brevianamid F | 85 | 87.9 | 88.6 | 95.2 | 81 | 89 |
| 3-Nitropropionic acid | 36.4 | 63 | 105.6 | 101 | 87 | 89.4 |  | Butyrolactone I | 139 | 120 | 125 | 125 | 125 | 125 |
| Abscisic qual | 123 | 120 | 120 | 120 | 120 | 120 |  | Chanoclavin | 50.5 | 57.9 | 55.3 | 29.4 | 69.5 | 83.5 |
| Aflatoxin B1 | 47 | 71.1 | 65.9 | 94.8 | 77.2 | 89.1 |  | Chloramphenicol | 90 | 74.2 | 100 | 99 | 103 | 113 |
| Aflatoxin B2 | 40 | 66.5 | 65.4 | 91.4 | 72.5 | 85.8 |  | Chrysogin | 85.3 | 82.9 | 88.2 | 85 | 85 | 85 |
| Aflatoxin G1 | 53 | 61.5 | 68.8 | 93.7 | 70.8 | 107.3 |  | Chrysophanol | 105 | 67.5 | 77.9 | 112 | 100 | 125 |
| Aflatoxin G2 | 68 | 61.9 | 73.8 | 89.7 | 83.8 | 100.9 |  | Citreoviridin | 87 | 91.1 | 92.3 | 85 | 85 | 78.9 |
| Aflatoxin M1 | 58.5 | 62.8 | 80.3 | 96.8 | 83 | 92.4 |  | Citrinin | 142 | 55.3 | 42.1 | 82.3 | 22.4 | 50 |
| Agroclavine | 85.5 | 68.1 | 62.3 | 82.8 | 51.7 | 81.5 |  | Cochlioquinone A | 65.2 | 24.3 | 40 | 40 | 40 | 40 |
| alpha-Zearalenol | 88.2 | 109 | 76.9 | 111 | 119 | 123 |  | Culmorin | 100 | 76.3 | 101.9 | 109.6 | 101.8 | 94.6 |
| Alternariol (AOH) | 84.1 | 104 | 87.8 | 113 | 114 | 105.7 |  | Curvularin | 95.8 | 133 | 97.2 | 115 | 105 | 125 |
| AOHmethylether | 94.8 | 104 | 90.4 | 110 | 104 | 111.5 |  | cyclo(L-Pro-L-Tyr) | 100 | 100 | 100 | 100 | 100 | 100 |
| Altertoxin-I | 95.5 | 117 | 69.1 | 97.3 | 86.8 | 104 |  | Cyclopiazonsäure | 160 | 150 | 142 | 150 | 150 | 155 |
| Andrastin A | 70 | 70 | 66.7 | 70 | 70 | 70 |  | Cytochalasin B | 101.5 | 77.8 | 90.9 | 99 | 95.4 | 91 |
| Antibiotic Y | 95.6 | 100 | 102.2 | 100 | 100 | 100 |  | Cytochalasin H | 97.2 | 83.2 | 92.9 | 91.5 | 90.3 | 95 |
| Apicidin | 114 | 96.2 | 75.3 | 112 | 99.2 | 100 |  | Cytochalasin J | 84.3 | 77.9 | 88.9 | 102.5 | 92.4 | 87.9 |
| Asperglaucide | 75.5 | 92.9 | 90 | 90 | 90 | 90 |  | Dehydrocurvularin | 69.4 | 79.8 | 90 | 100 | 100 | 90 |
| Aspterric acid | 84.8 | 85.4 | 77.9 | 92.5 | 96.2 | 85.1 |  | Deoxyfusapyron | 72 | 78.4 | 98.7 | 100 | 100 | 100 |
| Aurofusarin | 149 | 30.1 | 69.4 | 92.4 | 55.8 | 59.1 |  | Deoxynivalenol | 119 | 30.6 | 99.9 | 93.3 | 81.7 | 109.4 |
| Averantin | 47.6 | 68.1 | 87.4 | 84.2 | 81.2 | 70.8 |  | Destruxin A | 86.3 | 81.4 | 85 | 85 | 85 | 85 |
| Averufanin | 74 | 48.2 | 75.3 | 86.6 | 66.8 | 69.7 |  | Diacetoxyscirpenol | 85.8 | 81.2 | 75.9 | 101.1 | 91.5 | 90 |
| Averufin | 76.4 | 46.6 | 76.6 | 92 | 57.3 | 79.4 |  | DON-3-glucoside | 53.3 | 22.5 | 88.1 | 93 | 75 | 71 |
| Beauvericin | 100 | 108 | 110 | 109.1 | 106.1 | 93.4 |  | Elymoclavine | 82.7 | 68.4 | 75 | 75 | 75 | 70.6 |
| beta-Zearalenol | 86 | 109 | 83 | 109 | 99.9 | 117.6 |  | Elymoclavine-Fructoside | 30.4 | 51.8 | 59.8 | 50 | 50 | 39.5 |
| Bikav qual | 82.4 | 68 | 95.7 | 100.6 | 45.6 | 80 |  | Emodin | 94.5 | 102 | 75.1 | 102 | 86.2 | 93.7 |

**Table S1** Cont’d

| Metabolite | Recovery (%) | | | | | |  | Metabolite | Recovery (%) | | | | | |
| --- | --- | --- | --- | --- | --- | --- | --- | --- | --- | --- | --- | --- | --- | --- |
|  | Feed | GNC^b^ | Maize | Rice | Millet | Wheat |  |  | Feed | GNC^b^ | Maize | Rice | Millet | Wheat |
| Enniatin A | 83 | 99.8 | 103 | 111.3 | 89.8 | 105 |  | Fumonisin B1 (FB_1_) | 46.7 | 56.8 | 85.1 | 74 | 75 | 56.1 |
| Enniatin A1 | 89 | 97.3 | 101 | 103 | 89 | 98.1 |  | Fumonisin B2 | 53.7 | 65.6 | 86 | 86 | 75 | 75.4 |
| Enniatin B | 91.2 | 87.9 | 94.5 | 101.6 | 93.2 | 98.4 |  | Fumonisin B3 | 64.8 | 73.1 | 91.3 | 80 | 75 | 77.2 |
| Enniatin B1 | 90.5 | 90.2 | 96 | 100.6 | 91.7 | 95.7 |  | Fusapyron | 80.5 | 69.9 | 108.8 | 100 | 100 | 100 |
| Enniatin B2 | 93.4 | 88.7 | 98 | 104.4 | 89.3 | 93.9 |  | Fusaric acid | 79.3 | 73.7 | 77.7 | 82.1 | 33.5 | 75.4 |
| Enniatin B3 | 85.7 | 84.9 | 72 | 105 | 97.7 | 98 |  | Fusarinolic acid | 79.3 | 73.7 | 77.7 | 82.1 | 33.5 | 75.4 |
| Equisetin | 250 | 175 | 152.3 | 125 | 194 | 254 |  | Geldanamycin | 119 | 103 | 47 | 100 | 100 | 112 |
| Equisetin qual | 250 | 175 | 152.3 | 125 | 194 | 254 |  | Griseofulvin | 80.4 | 73.2 | 88.2 | 100.2 | 100.8 | 90.9 |
| Ergocornine | 84.6 | 77.4 | 55.7 | 75 | 75 | 75.5 |  | Harzianopyridine | 93.3 | 75.3 | 85 | 85 | 85 | 85 |
| Ergocorninin | 72.3 | 76.4 | 52.8 | 75 | 75 | 76.9 |  | Helvolic acid | 104.9 | 103.5 | 104.8 | 112 | 112 | 125 |
| Ergocristine | 77 | 113 | 56.3 | 75 | 75 | 75.4 |  | HT-2 toxin | 91.1 | 86.5 | 96.6 | 101 | 100 | 95.1 |
| Ergocristinine | 53.8 | 50.5 | 44.2 | 50 | 50 | 62.9 |  | Hydrolysed FB_1_ | 66.6 | 76.1 | 76.1 | 75 | 75 | 78.4 |
| Ergocryptine | 48 | 92.1 | 43.5 | 75 | 75 | 66.7 |  | Infectopyron | 84.1 | 74.7 | 80 | 80 | 80 | 80 |
| Ergocryptinine | 48.8 | 92.9 | 49 | 75 | 75 | 82.4 |  | Integracin A | 83.5 | 49.9 | 85 | 85 | 85 | 85 |
| Ergometrin | 126 | 135 | 70 | 70 | 70 | 45.4 |  | Integracin B | 61.1 | 89.4 | 85 | 85 | 85 | 85 |
| Ergometrinin | 62.1 | 96.6 | 60 | 60 | 60 | 106 |  | Kojic acid | 77.3 | 79.5 | 94.2 | 84.4 | 47.2 | 75 |
| Ergosin | 50 | 50 | 57.6 | 50 | 50 | 30.3 |  | Linamarin | 71.9 | 69.9 | 100 | 108 | 95.3 | 132.3 |
| Ergosinin | 22.4 | 34.8 | 35 | 40 | 40 | 40.5 |  | Lotaustralin | 90.4 | 64 | 100 | 111 | 82.4 | 116 |
| Ergotamine | 30 | 30 | 26.4 | 30 | 30 | 29.8 |  | Macrosporin | 143 | 142 | 107 | 128 | 128 | 150.2 |
| Ergotaminine | 30.5 | 34.4 | 75.5 | 30 | 30 | 36.8 |  | Malformin A | 103 | 83.9 | 100 | 100 | 100 | 100 |
| FB4 qual | 100 | 100 | 100 | 100 | 100 | 100 |  | Malformin C | 106 | 85.1 | 103.8 | 104 | 98.6 | 129 |
| Festuclavine | 75.4 | 84.1 | 64.2 | 81.9 | 56.8 | 85.3 |  | Meleagrin | 84.7 | 110 | 136 | 130 | 130 | 154 |
| Fumigaclavine C | 100 | 100 | 100 | 100 | 100 | 100 |  | Methylsulochrin | 94.2 | 114 | 88.2 | 103 | 107 | 113 |
| Fumiquinazolin A | 100 | 100 | 136 | 100 | 100 | 100 |  | Mevinolin | 85.9 | 95.7 | 113 | 100 | 100 | 94 |
| Fumiquinazolin D | 100 | 100 | 100 | 100 | 100 | 100 |  | Monactin | 88.1 | 91.2 | 94.3 | 114 | 111 | 88 |
| Fumitremorgin C | 84.8 | 89.8 | 110.7 | 90 | 90 | 84.7 |  | Moniliformin | 98.1 | 85 | 75.3 | 82.8 | 73.5 | 97.1 |

**Table S1** Cont’d

| Metabolite | Recovery (%) | | | | | |  | Metabolite | Recovery (%) | | | | | |
| --- | --- | --- | --- | --- | --- | --- | --- | --- | --- | --- | --- | --- | --- | --- |
|  | Feed | GNC^b^ | Maize | Rice | Millet | Wheat |  |  | Feed | GNC^b^ | Maize | Rice | Millet | Wheat |
| Monoacetoxyscirpenol | 76.4 | 61.5 | 91.2 | 99 | 98.2 | 82.5 |  | Radicicol | 136 | 122 | 127.2 | 121 | 171 | 155 |
| Monocerin | 96.1 | 76.8 | 90 | 103.7 | 100.4 | 97.1 |  | Rugulosin | 144 | 372 | 530.6 | 400 | 400 | 400 |
| Nidurufin | 103 | 117 | 94.6 | 114 | 115 | 131 |  | Rugulovasine A | 41 | 53.1 | 50 | 50 | 50 | 50 |
| Nivalenol | 33.1 | 21.1 | 72.8 | 85.5 | 81.7 | 77.3 |  | Rugulusovin | 87.7 | 73.5 | 87.5 | 85 | 85 | 85 |
| Nonactin | 81.5 | 83.8 | 88 | 106 | 105 | 96.3 |  | Secalonic acid D | 99.2 | 103 | 101.5 | 111 | 99.2 | 106 |
| Norsolorinic acid | 57 | 38.4 | 156.4 | 102.8 | 101.8 | 132 |  | Siccanol | 100 | 100 | 100 | 100 | 100 | 100 |
| Ochratoxin A | 83.8 | 71.8 | 92.9 | 101.3 | 84.4 | 93.3 |  | Skyrin | 75 | 64.7 | 74.2 | 56.6 | 71.6 | 32.3 |
| Ochratoxin B | 80.3 | 72.8 | 95.3 | 95 | 95 | 89.8 |  | Sterigmatocystin | 87.5 | 65.8 | 97.4 | 103.8 | 94.2 | 100 |
| O-Methylsterigmatocystin | 77.7 | 75.5 | 79.6 | 85 | 85 | 92.3 |  | T2-toxin | 85 | 88.7 | 71.8 | 100.6 | 96.2 | 95.8 |
| Oxaline | 71.1 | 106 | 100 | 100 | 100 | 100 |  | Tentoxin | 122 | 135 | 96.8 | 102 | 90.2 | 111.7 |
| Oxidized Elymoclavine | 89.3 | 183 | 75.5 | 75 | 75 | 17.5 |  | Tenuazonic acid | 150 | 170 | 186.7 | 173 | 137 | 177.1 |
| Paxillin | 86.6 | 72.3 | 105.5 | 97.7 | 94.3 | 105.3 |  | Territrem B | 60.2 | 76 | 93.6 | 90 | 90 | 93.8 |
| Penicillide | 91.3 | 112 | 100 | 100 | 100 | 115 |  | Tryptophol | 90 | 83.1 | 123.4 | 98.7 | 79.8 | 98 |
| Pestalotin | 86.5 | 77.8 | 87.4 | 85 | 85 | 94.4 |  | Usnic acid | 149 | 87.8 | 100 | 100 | 100 | 100 |
| Pseurotin A | 121 | 88.3 | 177.6 | 130 | 130 | 129 |  | Versicolorin A | 126 | 125 | 101.4 | 113 | 114 | 132 |
| Pyripyropene A | 60.5 | 64.5 | 93.8 | 90 | 90 | 92.3 |  | Versicolorin C | 126 | 125 | 119 | 111 | 112 | 150 |
| Questiomycin A | 87.9 | 71.4 | 85 | 85 | 85 | 85 |  | Zearalenone | 99.8 | 100.1 | 95.8 | 106 | 113 | 130 |
| Quinocitrinine A | 65.2 | 75.5 | 70 | 70 | 70 | 70 |  | Zearalenone-sulfate | 129 | 107 | 95.7 | 103 | 106 | 97.8 |

^a^Other method performance characteristics (LOD for each matrix: feed, peanut, maize, rice, millet and wheat) are according to Ezekiel *et al*., (2012), Warth *et al*., (2012), Abia *et al*., (2013) and Abdus-Salaam *et al*., (2015).

^b^Peanut cake.

**Table S2** Overview of the occurrence levels of additional microbial metabolites in 30 poultry feed samples from Nigeria.

| Metabolites | %^a^ | Concentration (µg/kg) | | |  | Metabolites | %^a^ | Concentration (µg/kg) | | |
| --- | --- | --- | --- | --- | --- | --- | --- | --- | --- | --- |
|  |  | Min | Max | Mean |  |  |  | Min | Max | Mean |
| 15-Hydroxyculmorin | 6.7 | 202 | 258 | 230 |  | Chrysogin | 13.3 | 4.1 | 20 | 11 |
| 15-Hydroxyculmoron | 6.7 | 40 | 88 | 64 |  | Chrysophanol | 3.3 | 20 | 20 | 0.0 |
| 3-Nitropropionic acid | 90 | 5.6 | 388 | 60 |  | Citreoviridin | 6.7 | 9.2 | 15 | 12 |
| Abscisic qual | 3.3 | 127 | 126 | 126 |  | Cochlioquinone A | 3.3 | 146 | 146 | 0.0 |
| Agroclavine | 30 | 0.0 | 1,480 | 471 |  | Culmorin | 6.7 | 22 | 44 | 33 |
| Altertoxin-I | 6.7 | 0.6 | 2.7 | 1.7 |  | Curvularin | 70 | 0.2 | 18 | 4.3 |
| Andrastin A | 13.3 | 6,030^b^ | 325,000^b^ | 92,843 |  | cyclo(L-Pro-L-Tyr) | 100 | 12 | 1,630 | 336 |
| Antibiotic Y | 3.3 | 167 | 167 | 167 |  | Cytochalasin H | 10 | 30 | 61 | 45 |
| Apicidin | 6.7 | 1.0 | 2.9 | 2.0 |  | Deoxyfusapyron | 16.7 | 4.3 | 56 | 16 |
| Asperglaucide | 100 | 8.6 | 2,230 | 530 |  | Destruxin A | 6.7 | 1.1 | 1.8 | 1.4 |
| Aurofusarin | 20 | 13 | 306 | 82 |  | Elymoclavine (ECV) | 23.3 | 0.8 | 2,390 | 818 |
| Averantin | 6.7 | 1.1 | 2.9 | 2.0 |  | ECV-Fructoside | 13.3 | 109 | 942 | 561 |
| Averufanin | 33.3 | 0.2 | 4.8 | 1.0 |  | Emodin | 83.3 | 0.3 | 14 | 4.9 |
| Averufin | 70 | 1.3 | 31 | 7.1 |  | Enniatin A | 40 | 0.2 | 1.4 | 0.5 |
| Bikav qual | 86.7 | 18 | 219 | 93 |  | Enniatin A1 | 53.3 | 1.0 | 12 | 3.6 |
| Bis(methylthio) gliotoxin | 13.3 | 7.9 | 49 | 25 |  | Enniatin B | 56.7 | 0.1 | 33 | 6.0 |
| Brevianamid F | 83.3 | 8.5 | 255 | 76 |  | Enniatin B1 | 56.7 | 0.4 | 36 | 9.0 |
| Chanoclavin | 40 | 0.3 | 242 | 48 |  | Enniatin B2 | 13.3 | 0.2 | 1.6 | 0.6 |
| Chloramphenicol | 20 | 0.1 | 0.8 | 0.3 |  | Enniatin B3 | 10 | 0.0 | 0.0 | 0.0 |
| Metabolites | %^a^ | Concentration (µg/kg) | | |  | Metabolites | %^a^ | Concentration (µg/kg) | | |
|  |  | Min | Max | Mean |  |  |  | Min | Max | Mean |
| Equisetin | 100 | 0.6 | 182 | 20 |  | Fusarinolic acid | 70 | 144 | 17,900 | 2,487 |
| Equisetin qual | 23.3 | 1.5 | 11 | 4.0 |  | Geldanamycin | 10 | 0.5 | 2.3 | 1.1 |
| Ergocornine | 10 | 0.7 | 16 | 8.3 |  | Harzianopyridine | 6.7 | 0.3 | 0.7 | 0.5 |
| Ergocorninin | 16.7 | 0.4 | 7.9 | 3.1 |  | Helvolic acid | 6.7 | 120 | 134 | 127 |
| Ergocristine | 33.3 | 1.1 | 35 | 12 |  | Infectopyron | 43.3 | 19 | 431 | 112 |
| Ergocristinine | 26.7 | 0.7 | 13 | 5.5 |  | Kojic acid | 93.3 | 89 | 10,990 | 2,221 |
| Ergocryptine | 20 | 3.6 | 38 | 13 |  | Linamarin | 13.3 | 96 | 5,610 | 1,572 |
| Ergocryptinine | 33.3 | 0.6 | 14 | 3.3 |  | Lotaustralin | 33.3 | 6.2 | 522 | 64 |
| Ergometrin | 23.3 | 0.1 | 0.8 | 0.3 |  | Macrosporin | 93.3 | 0.1 | 26 | 3.6 |
| Ergometrinin | 20 | 6.9 | 88 | 29 |  | Malformin A | 46.7 | 0.2 | 6.0 | 0.9 |
| Ergosin | 20 | 1.7 | 11 | 6.2 |  | Malformin C | 36.7 | 0.1 | 0.5 | 0.2 |
| Ergosinin | 23.3 | 0.7 | 4.6 | 2.0 |  | Methylsulochrin | 20 | 0.2 | 7.3 | 1.7 |
| Ergotamine | 23.3 | 1.8 | 20 | 7.8 |  | Monactin | 10 | 0.1 | 0.9 | 0.5 |
| Ergotaminine | 26.7 | 0.9 | 13 | 4.0 |  | Monoacetoxyscirpenol | 26.7 | 2.5 | 20 | 6.2 |
| Festuclavine | 33.3 | 0.2 | 91 | 23 |  | Monocerin | 90 | 0.7 | 150 | 22 |
| Fumiquinazolin A | 10 | 2,400^b^ | 179,000^b^ | 61,355 |  | Nidurufin | 40 | 0.0 | 0.7 | 0.2 |
| Fumiquinazolin D | 3.3 | 54,400^b^ | 54,400^b^ | 0.0 |  | Nonactin | 10 | 0.3 | 1.2 | 0.6 |
| Fumitremorgin C | 3.3 | 3.1 | 3.1 | 0.0 |  | Norsolorinic acid | 26.7 | 0.4 | 5.5 | 1.8 |
| Fusapyron | 3.3 | 19 | 19 | 0.0 |  | O-MethylSTER | 36.7 | 0.2 | 7.5 | 1.6 |
| Fusaric acid | 26.7 | 499 | 2,630 | 946 |  | Oxaline | 3.3 | 0.1 | 0.1 | 0.1 |
| Metabolites | %^a^ | Concentration (µg/kg) | | |  | Metabolites | %^a^ | Concentration (µg/kg) | | |
|  |  | Min | Max | Mean |  |  |  | Min | Max | Mean |
| Oxidized ECV | 13.3 | 110 | 514 | 265 |  | Secalonic acid D | 6.7 | 41 | 45 | 43 |
| Pestalotin | 3.3 | 4.4 | 4.4 | 0.0 |  | Siccanol | 66.7 | 2,400^b^ | 106,000^b^ | 18,288 |
| Pseurotin A | 3.3 | 21 | 21 | 0.0 |  | Skyrin | 26.7 | 1.1 | 19.5 | 6.4 |
| Pyripyropene A | 13.3 | 1.8 | 15 | 5.5 |  | Sterigmatocystin (STER) | 46.7 | 0.3 | 4.9 | 1.3 |
| Questiomycin A | 50 | 6.6 | 51 | 19 |  | Tentoxin | 60 | 0.1 | 21 | 3.5 |
| Quinocitrinine A | 86.7 | 2.3 | 1,370 | 109 |  | Tryptophol | 80 | 31 | 538 | 258 |
| Radicicol | 30 | 1.3 | 238 | 34 |  | Usnic acid | 3.3 | 0.2 | 0.2 | 0.0 |
| Rugulosin | 3.3 | 5.7 | 5.7 | 0.0 |  | Versicolorin A | 46.7 | 0.1 | 2.0 | 0.5 |
| Rugulovasine A | 6.7 | 28 | 55 | 42 |  | Versicolorin C | 66.7 | 0.2 | 13 | 1.9 |
| Rugulusovin | 70 | 4.4 | 48 | 13 |  |  |  |  |  |  |

^a^Incidence of contamination expressed in percentage

^b^Values denote peak areas (no quantitative standard available)

**Table S3** Occurrence levels of additional microbial metabolites in 44 cereal and nut ingredients for poultry feed in Nigeria.

| Metabolites | Maize (*n^a^*= 17; *n^b^*= 84) | | | | Peanut cake (*n^a^*= 11; *n^b^*= 68) | | | | Wheat offal (*n^a^*= 10; *n^b^*= 106) | | | | Other cereals (*n^a^*= 6; *n^b^*= 39) | | | |
| --- | --- | --- | --- | --- | --- | --- | --- | --- | --- | --- | --- | --- | --- | --- | --- | --- |
|  | %^c^ | Concentration (µg/kg) | | | %^c^ | Concentration (µg/kg) | | | %^c^ | Concentration (µg/kg) | | | %^c^ | Concentration (µg/kg) | | |
|  |  | Min | Max | Mean |  | Min | Max | Mean |  | Min | Max | Mean |  | Min | Max | Mean |
| 15-Hydroxyculmoron | 5.9 | 7.4 | 7.4 | 0.0 | 0.0 | 0.0 | 0.0 | 0.0 | 10 | 130 | 130 | 0.0 | 0.0 | 0.0 | 0.0 | 0.0 |
| 3-Nitropropionic acid | 47.1 | 4.1 | 25 | 11 | 100 | 2.3 | 585 | 1220 | 30 | 2.8 | 9.2 | 6.4 | 50.0 | 4.1 | 389 | 154 |
| Abscisic qual | 0.0 | 0.0 | 0.0 | 0.0 | 0.0 | 0.0 | 0.0 | 0.0 | 0.0 | 0.0 | 0.0 | 0.0 | 16.7 | 302 | 302 | 0.0 |
| Agroclavine | 23.5 | 2.0 | 14 | 8.8 | 18.2 | 0.5 | 9.4 | 5.0 | 40 | 0.3 | 1.8 | 0.7 | 33.3 | 4,054 | 6,395 | 5,225 |
| alpha-Zearalenol | 0.0 | 0.0 | 0.0 | 0.0 | 0.0 | 0.0 | 0.0 | 0.0 | 20 | 5.1 | 5.4 | 5.2 | 0.0 | 0.0 | 0.0 | 0.0 |
| Altertoxin-I | 0.0 | 0.0 | 0.0 | 0.0 | 0.0 | 0.0 | 0.0 | 0.0 | 20 | 2.1 | 2.2 | 2.2 | 0.0 | 0.0 | 0.0 | 0.0 |
| Andrastin A | 5.9 | 3,060^d^ | 3,060^d^ | 0.0 | 18.2 | 3,860^d^ | 141,000^d^ | 72,020 | 10 | 96,000^d^ | 96,000^d^ | 0.0 | 0.0 | 0.0 | 0.0 | 0.0 |
| Antibiotic Y | 0.0 | 0.0 | 0.0 | 0.0 | 0.0 | 0.0 | 0.0 | 0.0 | 20 | 514 | 639 | 577 | 0.0 | 0.0 | 0.0 | 0.0 |
| Apicidin | 0.0 | 0.0 | 0.0 | 0.0 | 0.0 | 0.0 | 0.0 | 0.0 | 30 | 0.7 | 6.2 | 2.7 | 0.0 | 0.0 | 0.0 | 0.0 |
| Asperglaucide | 94.1 | 1.9 | 8,200 | 1,813 | 100 | 60 | 1,068 | 303 | 90 | 28 | 4,590 | 686 | 83.3 | 4.3 | 263 | 114 |
| Aspterric acid | 5.9 | 92 | 92 | 0.0 | 0.0 | 0.0 | 0.0 | 0.0 | 0.0 | 0.0 | 0.0 | 0.0 | 0.0 | 0.0 | 0.0 | 0.0 |
| Aurofusarin | 0.0 | 0.0 | 0.0 | 0.0 | 0.0 | 0.0 | 0.0 | 0.0 | 30 | 62 | 3,160 | 1,698 | 0.0 | 0.0 | 0.0 | 0.0 |
| Averantin | 5.9 | 7.6 | 7.6 | 7.6 | 72.7 | 0.2 | 9.7 | 2.2 | 20 | 1.1 | 1.8 | 1.5 | 0.0 | 0.0 | 0.0 | 0.0 |
| Averufanin | 23.5 | 0.3 | 6.2 | 2.3 | 81.8 | 0.9 | 51.4 | 9.1 | 20 | 2.2 | 2.4 | 2.3 | 0.0 | 0.0 | 0.0 | 0.0 |
| Averufin | 29.4 | 2.4 | 60 | 17 | 90.9 | 17 | 353 | 90 | 20 | 33 | 37 | 34.9 | 16.7 | 0.7 | 0.7 | 0.0 |
| beta-Zearalenol | 0.0 | 0.0 | 0.0 | 0.0 | 0.0 | 0.0 | 0.0 | 0.0 | 10 | 5.4 | 5.4 | 0.0 | 0.0 | 0.0 | 0.0 | 0.0 |
| Butyrolactone I | 0.0 | 0.0 | 0.0 | 0.0 | 0.0 | 0.0 | 0.0 | 0.0 | 20 | 101 | 130 | 115 | 0.0 | 0.0 | 0.0 | 0.0 |

**Table S3** Continued

| Metabolites | Maize (*n^a^*= 17; *n^b^*= 84) | | | | Peanut cake (*n^a^*= 11; *n^b^*= 68) | | | | | Wheat offal (*n^a^*= 10; *n^b^*= 106) | | | | | Other cereals (*n^a^*= 6; *n^b^*= 39) | | | | |
| --- | --- | --- | --- | --- | --- | --- | --- | --- | --- | --- | --- | --- | --- | --- | --- | --- | --- | --- | --- |
|  | %^c^ | Concentration (µg/kg) | | | | %^c^ | Concentration (µg/kg) | | | | %^c^ | Concentration (µg/kg) | | | | %^c^ | Concentration (µg/kg) | | |
|  |  | Min | Max | Mean | |  | Min | Max | Mean | |  | Min | Max | Mean | |  | Min | Max | Mean |
| Chanoclavin | 47.1 | 0.1 | 1.5 | 0.4 | | 9.1 | 0.6 | 0.6 | 0.0 | | 50 | 0.5 | 4.1 | 1.9 | | 33.3 | 851 | 1,390 | 1,120 |
| Chloramphenicol | 23.5 | 0.1 | 0.4 | 0.2 | | 18.2 | 1.6 | 3.7 | 2.7 | | 40 | 0.1 | 0.7 | 0.3 | | 33.3 | 0.1 | 0.2 | 0.1 |
| Chrysogin | 29.4 | 4.2 | 25 | 12 | | 0.0 | 0.0 | 0.0 | 0.0 | | 30 | 9.6 | 16 | 12 | | 0.0 | 0.0 | 0.0 | 0.0 |
| Chrysophanol | 0.0 | 0.0 | 0.0 | 0.0 | | 0.0 | 0.0 | 0.0 | 0.0 | | 20 | 11 | 13 | 12 | | 0.0 | 0.0 | 0.0 | 0.0 |
| Citreoviridin | 23.5 | 2.7 | 159 | 66 | | 18.2 | 4.4 | 7.8 | 6.1 | | 20 | 88 | 139 | 113 | | 0.0 | 0.0 | 0.0 | 0.0 |
| Cochlioquinone A | 0.0 | 0.0 | 0.0 | 0.0 | | 0.0 | 0.0 | 0.0 | 0.0 | | 10 | 180 | 180 | 0.0 | | 0.0 | 0.0 | 0.0 | 0.0 |
| Culmorin | 0.0 | 0.0 | 0.0 | 0.0 | | 0.0 | 0.0 | 0.0 | 0.0 | | 30 | 22 | 108 | 62 | | 0.0 | 0.0 | 0.0 | 0.0 |
| Curvularin | 29.4 | 1.3 | 21 | 5.5 | | 81.8 | 0.5 | 46 | 6.9 | | 80 | 1.6 | 89 | 24 | | 0.0 | 0.0 | 0.0 | 0.0 |
| cyclo(L-Pro-L-Tyr) | 100 | 3.3 | 130 | 41 | | 100 | 3.3 | 699 | 232 | | 100 | 16 | 102 | 46 | | 66.7 | 2.7 | 495 | 136 |
| Cyclopiazonic acid | 5.9 | 98 | 98 | 0.0 | | 27.3 | 34 | 204 | 93 | | 0.0 | 0.0 | 0.0 | 0.0 | | 0.0 | 0.0 | 0.0 | 0.0 |
| Cytochalasin B | 0.0 | 0.0 | 0.0 | 0.0 | | 0.0 | 0.0 | 0.0 | 0.0 | | 20 | 62 | 64 | 63 | | 0.0 | 0.0 | 0.0 | 0.0 |
| Cytochalasin H | 0.0 | 0.0 | 0.0 | 0.0 | | 9.1 | 416 | 416 | 0.0 | | 0.0 | 0.0 | 0.0 | 0.0 | | 16.7 | 55 | 55 | 0.0 |
| Cytochalasin J | 5.9 | 10 | 10 | 0.0 | | 36.4 | 9.6 | 776 | 204 | | 0.0 | 0.0 | 0.0 | 0.0 | | 16.7 | 100 | 100 | 0.0 |
| Dehydrocurvularin | 0.0 | 0.0 | 0.0 | 0.0 | | 0.0 | 0.0 | 0.0 | 0.0 | | 20 | 8.9 | 39 | 24 | | 0.0 | 0.0 | 0.0 | 0.0 |
| Deoxyfusapyron | 29.4 | 2.0 | 29 | 8.2 | | 0.0 | 0.0 | 0.0 | 0.0 | | 0.0 | 0.0 | 0.0 | 0.0 | | 0.0 | 0.0 | 0.0 | 0.0 |
| Destruxin A | 0.0 | 0.0 | 0.0 | 0.0 | | 9.1 | 1.5 | 1.5 | 0.0 | | 20 | 7.5 | 11 | 9.1 | | 0.0 | 0.0 | 0.0 | 0.0 |
| Diacetoxyscirpenol | 5.9 | 1.8 | 1.8 | 0.0 | | 0.0 | 0.0 | 0.0 | 0.0 | | 0.0 | 0.0 | 0.0 | 0.0 | | 0.0 | 0.0 | 0.0 | 0.0 |
| DON-3-glucoside | 0.0 | 0.0 | 0.0 | 0.0 | | 0.0 | 0.0 | 0.0 | 0.0 | | 20 | 116 | 170 | 143 | | 0.0 | 0.0 | 0.0 | 0.0 |

**Table S3** Continued

| Metabolites | Maize (*n^a^*= 17; *n^b^*= 84) | | | | Peanut cake (*n^a^*= 11; *n^b^*= 68) | | | | Wheat offal (*n^a^*= 10; *n^b^*= 106) | | | | Other cereals (*n^a^*= 6; *n^b^*= 39) | | | |
| --- | --- | --- | --- | --- | --- | --- | --- | --- | --- | --- | --- | --- | --- | --- | --- | --- |
|  | %^c^ | Concentration (µg/kg) | | | %^c^ | Concentration (µg/kg) | | | %^c^ | Concentration (µg/kg) | | | %^c^ | Concentration (µg/kg) | | |
|  |  | Min | Max | Mean |  | Min | Max | Mean |  | Min | Max | Mean |  | Min | Max | Mean |
| Elymoclavine (ECV) | 23.5 | 2.3 | 15 | 9.0 | 9.1 | 14 | 14 | 0.0 | 10 | 2.1 | 2.1 | 0.0 | 33.3 | 6,580 | 7,510 | 7,043 |
| ECV-Fructoside | 0.0 | 0.0 | 0.0 | 0.0 | 0.0 | 0.0 | 0.0 | 0.0 | 0.0 | 0.0 | 0.0 | 0.0 | 33.3 | 2,170 | 4,950 | 3,564 |
| Emodin | 76.5 | 0.1 | 23 | 2.9 | 100 | 0.5 | 6.0 | 1.3 | 100 | 1.9 | 53 | 15 | 66.7 | 2.1 | 3.7 | 2.7 |
| Enniatin A | 11.8 | 0.0 | 0.0 | 0.0 | 0.0 | 0.0 | 0.0 | 0.0 | 70 | 0.9 | 3.9 | 2.4 | 0.0 | 0.0 | 0.0 | 0.0 |
| Enniatin A1 | 11.8 | 0.6 | 0.6 | 0.6 | 9.1 | 0.3 | 0.3 | 0.3 | 70 | 7.4 | 51 | 24 | 0.0 | 0.0 | 0.0 | 0.0 |
| Enniatin B | 23.5 | 0.0 | 0.6 | 0.3 | 36.4 | 0.0 | 0.2 | 0.1 | 70 | 11 | 90 | 35 | 16.7 | 0.0 | 0.0 | 0.0 |
| Enniatin B1 | 23.5 | 0.1 | 1.2 | 0.6 | 18.2 | 0.1 | 0.2 | 0.1 | 70 | 17 | 149 | 58 | 0.0 | 0.0 | 0.0 | 0.0 |
| Enniatin B2 | 0.0 | 0.0 | 0.0 | 0.0 | 0.0 | 0.0 | 0.0 | 0.0 | 40 | 0.4 | 1.9 | 1.2 | 0.0 | 0.0 | 0.0 | 0.0 |
| Enniatin B3 | 0.0 | 0.0 | 0.0 | 0.0 | 0.0 | 0.0 | 0.0 | 0.0 | 40 | 0.0 | 0.0 | 0.0 | 0.0 | 0.0 | 0.0 | 0.0 |
| Equisetin | 88.2 | 0.4 | 105 | 23 | 100 | 1.3 | 95 | 17 | 90 | 0.4 | 11 | 2.8 | 100 | 0.2 | 9.3 | 2.1 |
| Equisetin qual | 5.9 | 1.8 | 1.8 | 0.0 | 18.2 | 1.4 | 2.2 | 1.8 | 0.0 | 0.0 | 0.0 | 0.0 | 16.7 | 0.1 | 0.1 | 0.1 |
| Ergocornine | 0.0 | 0.0 | 0.0 | 0.0 | 0.0 | 0.0 | 0.0 | 0.0 | 40 | 6.7 | 53 | 27 | 0.0 | 0.0 | 0.0 | 0.0 |
| Ergocorninin | 0.0 | 0.0 | 0.0 | 0.0 | 0.0 | 0.0 | 0.0 | 0.0 | 40 | 4.2 | 35 | 15 | 0.0 | 0.0 | 0.0 | 0.0 |
| Ergocristine | 0.0 | 0.0 | 0.0 | 0.0 | 0.0 | 0.0 | 0.0 | 0.0 | 70 | 0.9 | 73 | 50 | 0.0 | 0.0 | 0.0 | 0.0 |
| Ergocristinine | 0.0 | 0.0 | 0.0 | 0.0 | 0.0 | 0.0 | 0.0 | 0.0 | 60 | 6.6 | 30 | 22 | 0.0 | 0.0 | 0.0 | 0.0 |
| Ergocryptine | 5.9 | 2.0 | 2.0 | 0.0 | 0.0 | 0.0 | 0.0 | 0.0 | 50 | 11 | 137 | 43 | 0.0 | 0.0 | 0.0 | 0.0 |
| Ergocryptinine | 0.0 | 0.0 | 0.0 | 0.0 | 0.0 | 0.0 | 0.0 | 0.0 | 50 | 5.4 | 52 | 16 | 0.0 | 0.0 | 0.0 | 0.0 |
| Ergometrin | 0.0 | 0.0 | 0.0 | 0.0 | 0.0 | 0.0 | 0.0 | 0.0 | 50 | 0.7 | 8.6 | 4.1 | 0.0 | 0.0 | 0.0 | 0.0 |

**Table S3** Continued

| Metabolites | Maize (*n^a^*= 17; *n^b^*= 84) | | | | Peanut cake (*n^a^*= 11; *n^b^*= 68) | | | | | | Wheat offal (*n^a^*= 10; *n^b^*= 106) | | | | | | | | | Other cereals (*n^a^*= 6; *n^b^*= 39) | | | | | | |  |  |  |
| --- | --- | --- | --- | --- | --- | --- | --- | --- | --- | --- | --- | --- | --- | --- | --- | --- | --- | --- | --- | --- | --- | --- | --- | --- | --- | --- | --- | --- | --- |
|  | %^c^ | Concentration (µg/kg) | | |  | | Concentration (µg/kg) | | | | |  | | Concentration (µg/kg) | | | | | | |  | Concentration (µg/kg) | | | | | |  |  |
|  |  | Min | Max | Mean | | %^c^ | | Min | Max | Mean | | | %^c^ | | Min | Max | | | Mean | | %^c^ | | Min | Max | | Mean | | |  |
| Ergometrinin | 0.0 | 0.0 | 0.0 | 0.0 | | 0.0 | | 0.0 | 0.0 | 0.0 | | | 60 | | 7.8 | | 181 | 85 | | 0.0 | | | 0.0 | | 0.0 | 0.0 | | | |
| Ergosin | 0.0 | 0.0 | 0.0 | 0.0 | | 0.0 | | 0.0 | 0.0 | 0.0 | | | 60 | | 3.9 | | 72 | 47 | | 0.0 | | | 0.0 | | 0.0 | 0.0 | | | |
| Ergosinin | 0.0 | 0.0 | 0.0 | 0.0 | | 0.0 | | 0.0 | 0.0 | 0.0 | | | 60 | | 0.5 | | 23 | 10 | | 0.0 | | | 0.0 | | 0.0 | 0.0 | | | |
| Ergotamine | 0.0 | 0.0 | 0.0 | 0.0 | | 0.0 | | 0.0 | 0.0 | 0.0 | | | 60 | | 6.8 | | 89 | 50 | | 0.0 | | | 0.0 | | 0.0 | 0.0 | | | |
| Ergotaminine | 0.0 | 0.0 | 0.0 | 0.0 | | 0.0 | | 0.0 | 0.0 | 0.0 | | | 60 | | 1.1 | | 43 | 17 | | 0.0 | | | 0.0 | | 0.0 | 0.0 | | | |
| Festuclavine | 0.0 | 0.0 | 0.0 | 0.0 | | 9.1 | | 0.7 | 0.7 | 0.7 | | | 10 | | 1.5 | | 1.5 | 0.0 | | 33.3 | | | 220 | | 261 | 240 | | | |
| Fumiquinazolin A | 0.0 | 0.0 | 0.0 | 0.0 | | 0.0 | | 0.0 | 0.0 | 0.0 | | | 10 | | 5,030^d^ | | 5,030^d^ | 0.0 | | 0.0 | | | 0.0 | | 0.0 | 0.0 | | | |
| Fusapyron | 5.9 | 26 | 26 | 0.0 | | 0.0 | | 0.0 | 0.0 | 0.0 | | | 0.0 | | 0.0 | | 0.0 | 0.0 | | 0.0 | | | 0.0 | | 0.0 | 0.0 | | | |
| Fusaric acid | 58.8 | 433 | 2,720 | 892 | | 9.1 | | 1,830 | 1,830 | 0.0 | | | 0.0 | | 0.0 | | 0.0 | 0.0 | | 0.0 | | | 0.0 | | 0.0 | 0.0 | | | |
| Fusarinolic acid | 88.2 | 339 | 12,100 | 3,729 | | 9.1 | | 1,630 | 1,630 | 0.0 | | | 10 | | 774 | | 774 | 0.0 | | 0.0 | | | 0.0 | | 0.0 | 0.0 | | | |
| Geldanamycin | 0.0 | 0.0 | 0.0 | 0.0 | | 0.0 | | 0.0 | 0.0 | 0.0 | | | 20 | | 0.6 | | 1.3 | 0.9 | | 16.7 | | | 0.3 | | 0.3 | 0.0 | | | |
| Griseofulvin | 5.9 | 3.2 | 3.2 | 0.0 | | 0.0 | | 0.0 | 0.0 | 0.0 | | | 0.0 | | 0.0 | | 0.0 | 0.0 | | 0.0 | | | 0.0 | | 0.0 | 0.0 | | | |
| Helvolic acid | 0.0 | 0.0 | 0.0 | 0.0 | | 0.0 | | 0.0 | 0.0 | 0.0 | | | 10 | | 19 | | 19 | 0.0 | | 0.0 | | | 0.0 | | 0.0 | 0.0 | | | |
| HT-2 toxin | 0.0 | 0.0 | 0.0 | 0.0 | | 0.0 | | 0.0 | 0.0 | 0.0 | | | 10 | | 33 | | 33 | 0.0 | | 0.0 | | | 0.0 | | 0.0 | 0.0 | | | |
| Infectopyron | 5.9 | 23 | 23 | 0.0 | | 0.0 | | 0.0 | 0.0 | 0.0 | | | 70 | | 365 | | 1,680 | 864 | | 0.0 | | | 0.0 | | 0.0 | 0.0 | | | |
| Integracin A | 0.0 | 0.0 | 0.0 | 0.0 | | 45.5 | | 0.2 | 0.5 | 0.4 | | | 0.0 | | 0.0 | | 0.0 | 0.0 | | 0.0 | | | 0.0 | | 0.0 | 0.0 | | | |
| Integracin B | 0.0 | 0.0 | 0.0 | 0.0 | | 18.2 | | 0.8 | 1.3 | 1.0 | | | 0.0 | | 0.0 | | 0.0 | 0.0 | | 0.0 | | | 0.0 | | 0.0 | 0.0 | | | |
| Kojic acid | 58.8 | 68 | 11,200 | 3,002 | | 100 | | 75 | 7,320 | 2,560 | | | 20 | | 156 | | 201 | 178 | | 66.7 | | | 203 | | 540 | 340 | | | |

**Table S3** Continued

| Metabolites | Maize (*n^a^*= 17; *n^b^*= 84) | | | | Peanut cake (*n^a^*= 11; *n^b^*= 68) | | | | Wheat offal (*n^a^*= 10; *n^b^*= 106) | | | | Other cereals (*n^a^*= 6; *n^b^*= 39) | | | |
| --- | --- | --- | --- | --- | --- | --- | --- | --- | --- | --- | --- | --- | --- | --- | --- | --- |
|  | %^c^ | Concentration (µg/kg) | | | %^c^ | Concentration (µg/kg) | | | %^c^ | Concentration (µg/kg) | | | %^c^ | Concentration (µg/kg) | | |
|  |  | Min | Max | Mean |  | Min | Max | Mean |  | Min | Max | Mean |  | Min | Max | Mean |
| Linamarin | 17.6 | 21 | 1,840 | 998 | 18.2 | 1,490 | 2,048 | 1,769 | 20 | 929 | 954 | 942 | 0.0 | 0.0 | 0.0 | 0.0 |
| Lotaustralin | 29.4 | 7.0 | 137 | 55 | 54.5 | 18 | 378 | 136 | 40 | 6.6 | 73 | 41 | 0.0 | 0.0 | 0.0 | 0.0 |
| Macrosporin | 82.4 | 0.1 | 16 | 4.1 | 27.3 | 0.4 | 0.9 | 0.6 | 100 | 0.7 | 29 | 11 | 83.3 | 4.2 | 13 | 6.9 |
| Malformin A | 23.5 | 0.2 | 1.8 | 1.0 | 54.5 | 0.6 | 27 | 5.3 | 30 | 0.7 | 1.4 | 1.1 | 0.0 | 0.0 | 0.0 | 0.0 |
| Malformin C | 23.5 | 0.3 | 0.8 | 0.5 | 0.0 | 0.0 | 0.0 | 0.0 | 20 | 0.2 | 0.2 | 0.2 | 0.0 | 0.0 | 0.0 | 0.0 |
| Meleagrin | 0.0 | 0.0 | 0.0 | 0.0 | 0.0 | 0.0 | 0.0 | 0.0 | 10 | 1.8 | 1.8 | 0.0 | 0.0 | 0.0 | 0.0 | 0.0 |
| Methylsulochrin | 0.0 | 0.0 | 0.0 | 0.0 | 0.0 | 0.0 | 0.0 | 0.0 | 20 | 2.2 | 4.8 | 3.5 | 0.0 | 0.0 | 0.0 | 0.0 |
| Monactin | 11.8 | 0.1 | 0.9 | 0.5 | 27.3 | 0.0 | 0.1 | 0.1 | 20 | 0.8 | 1.6 | 1.2 | 0.0 | 0.0 | 0.0 | 0.0 |
| Monoacetoxyscirpenol | 35.3 | 2.8 | 8.1 | 5.4 | 9.1 | 4.3 | 4.3 | 0.0 | 20 | 4.1 | 4.2 | 4.1 | 0.0 | 0.0 | 0.0 | 0.0 |
| Monocerin | 52.9 | 3.3 | 289 | 79 | 36.4 | 0.4 | 73 | 19 | 70 | 1.3 | 29 | 13 | 33.3 | 0.4 | 1.9 | 1.1 |
| Nidurufin | 17.6 | 0.1 | 0.3 | 0.2 | 81.8 | 0.3 | 3.3 | 1.6 | 20 | 0.4 | 0.5 | 0.4 | 0.0 | 0.0 | 0.0 | 0.0 |
| Nonactin | 5.9 | 0.3 | 0.3 | 0.0 | 0.0 | 0.0 | 0.0 | 0.0 | 10 | 0.6 | 0.6 | 0.0 | 0.0 | 0.0 | 0.0 | 0.0 |
| Norsolorinic acid | 17.6 | 0.3 | 3.4 | 1.8 | 100 | 0.2 | 43 | 8.3 | 20 | 7.9 | 12 | 10 | 0.0 | 0.0 | 0.0 | 0.0 |
| O-MethylSTER | 41.2 | 0.1 | 5.9 | 1.3 | 72.7 | 5.9 | 67 | 16 | 20 | 2.1 | 2.8 | 2.4 | 0.0 | 0.0 | 0.0 | 0.0 |
| Oxaline | 17.6 | 0.3 | 2.4 | 1.0 | 0.0 | 0.0 | 0.0 | 0.0 | 10 | 0.4 | 0.4 | 0.0 | 0.0 | 0.0 | 0.0 | 0.0 |
| Oxidized ECV | 0.0 | 0.0 | 0.0 | 0.0 | 0.0 | 0.0 | 0.0 | 0.0 | 0.0 | 0.0 | 0.0 | 0.0 | 33.3 | 2,890 | 3,220 | 3,054 |
| Paxillin | 5.9 | 85 | 85 | 0.0 | 0.0 | 0.0 | 0.0 | 0.0 | 0.0 | 0.0 | 0.0 | 0.0 | 0.0 | 0.0 | 0.0 | 0.0 |
| Penicillide | 0.0 | 0.0 | 0.0 | 0.0 | 9.1 | 5.6 | 5.6 | 0.0 | 0.0 | 0.0 | 0.0 | 0.0 | 0.0 | 0.0 | 0.0 | 0.0 |

**Table S3** Continued

| Metabolites | Maize (*n^a^*= 17; *n^b^*= 84) | | | | Peanut cake (*n^a^*= 11; *n^b^*= 68) | | | | Wheat offal (*n^a^*= 10; *n^b^*= 106) | | | | Other cereals (*n^a^*= 6; *n^b^*= 39) | | | |
| --- | --- | --- | --- | --- | --- | --- | --- | --- | --- | --- | --- | --- | --- | --- | --- | --- |
|  | %^c^ | Concentration (µg/kg) | | | %^c^ | Concentration (µg/kg) | | | %^c^ | Concentration (µg/kg) | | | %^c^ | Concentration (µg/kg) | | |
|  |  | Min | Max | Mean |  | Min | Max | Mean |  | Min | Max | Mean |  | Min | Max | Mean |
| Pestalotin | 29.4 | 4.0 | 28 | 17 | 0.0 | 0.0 | 0.0 | 0.0 | 0.0 | 0.0 | 0.0 | 0.0 | 0.0 | 0.0 | 0.0 | 0.0 |
| Pseurotin A | 5.9 | 0.9 | 0.9 | 0.0 | 0.0 | 0.0 | 0.0 | 0.0 | 0.0 | 0.0 | 0.0 | 0.0 | 0.0 | 0.0 | 0.0 | 0.0 |
| Pyripyropene A | 11.8 | 1.3 | 5.5 | 3.4 | 0.0 | 0.0 | 0.0 | 0.0 | 0.0 | 0.0 | 0.0 | 0.0 | 0.0 | 0.0 | 0.0 | 0.0 |
| Questiomycin A | 76.5 | 9.9 | 90 | 40 | 0.0 | 0.0 | 0.0 | 0.0 | 0.0 | 0.0 | 0.0 | 0.0 | 0.0 | 0.0 | 0.0 | 0.0 |
| Quinocitrinine A | 76.5 | 0.3 | 1,920 | 353 | 100 | 1.7 | 134 | 20 | 30 | 10 | 24 | 19 | 50 | 2.7 | 4.7 | 3.9 |
| Radicicol | 41.2 | 2.2 | 91 | 32 | 9.1 | 8.9 | 8.9 | 8.9 | 0.0 | 0.0 | 0.0 | 0.0 | 0.0 | 0.0 | 0.0 | 0.0 |
| Rugulosin | 0.0 | 0.0 | 0.0 | 0.0 | 0.0 | 0.0 | 0.0 | 0.0 | 10 | 3.2 | 3.2 | 0.0 | 0.0 | 0.0 | 0.0 | 0.0 |
| Rugulovasine A | 0.0 | 0.0 | 0.0 | 0.0 | 0.0 | 0.0 | 0.0 | 0.0 | 20 | 41 | 41 | 41 | 0.0 | 0.0 | 0.0 | 0.0 |
| Rugulusovin | 35.3 | 2.3 | 22 | 14 | 81.8 | 11.2 | 72 | 20 | 50 | 8.4 | 25 | 15 | 16.7 | 1.2 | 1.2 | 0.0 |
| Secalonic acid D | 0.0 | 0.0 | 0.0 | 0.0 | 0.0 | 0.0 | 0.0 | 0.0 | 30 | 21 | 168 | 88 | 0.0 | 0.0 | 0.0 | 0.0 |
| Siccanol | 52.9 | 2,040^d^ | 9,750^d^ | 4,628 | 81.8 | 1,313^d^ | 21,400^d^ | 4,940 | 70 | 2,220^d^ | 55,400^d^ | 19,200 | 16.7 | 3,950^d^ | 3,950^d^ | 0.0 |
| Skyrin | 41.2 | 1.7 | 13 | 5.3 | 0.0 | 0.0 | 0.0 | 0.0 | 20 | 0.7 | 36 | 18 | 33.3 | 3.1 | 3.4 | 3.2 |
| Sterigmatocystin | 41.2 | 0.3 | 1.7 | 0.7 | 81.8 | 1.1 | 23 | 5.0 | 40 | 0.3 | 53 | 25 | 16.7 | 0.6 | 0.6 | 0.6 |
| T2-toxin | 0.0 | 0.0 | 0.0 | 0.0 | 0.0 | 0.0 | 0.0 | 0.0 | 10 | 11 | 11 | 0.0 | 0.0 | 0.0 | 0.0 | 0.0 |
| Tentoxin | 5.9 | 0.3 | 0.3 | 0.0 | 0.0 | 0.0 | 0.0 | 0.0 | 90 | 2.1 | 37 | 20 | 33.3 | 2.8 | 2.8 | 2.8 |
| Tryptophol | 41.2 | 11 | 103 | 38 | 63.6 | 60 | 126 | 94 | 100 | 28 | 2,950 | 1,210 | 16.7 | 481 | 481 | 0.0 |
| Usnic acid | 0.0 | 0.0 | 0.0 | 0.0 | 36.4 | 0.4 | 0.8 | 0.6 | 0.0 | 0.0 | 0.0 | 0.0 | 0.0 | 0.0 | 0.0 | 0.0 |

**Table S3** Continued

| Metabolites | Maize (*n^a^*= 17; *n^b^*= 84) | | | | Peanut cake (*n^a^*= 11; *n^b^*= 68) | | | | Wheat offal (*n^a^*= 10; *n^b^*= 106) | | | | Other cereals (*n^a^*= 6; *n^b^*= 39) | | | |
| --- | --- | --- | --- | --- | --- | --- | --- | --- | --- | --- | --- | --- | --- | --- | --- | --- |
|  | %^c^ | Concentration (µg/kg) | | | %^c^ | Concentration (µg/kg) | | | %^c^ | Concentration (µg/kg) | | | %^c^ | Concentration (µg/kg) | | |
|  |  | Min | Max | Mean |  | Min | Max | Mean |  | Min | Max | Mean |  | Min | Max | Mean |
| Versicolorin A | 35.3 | 0.1 | 2.0 | 0.7 | 90.9 | 0.6 | 14 | 3.2 | 20 | 0.9 | 1.0 | 0.9 | 0.0 | 0.0 | 0.0 | 0.0 |
| Versicolorin C | 47.1 | 0.2 | 15 | 3.3 | 90.9 | 2.4 | 84 | 16 | 30 | 0.1 | 8.2 | 5.0 | 0.0 | 0.0 | 0.0 | 0.0 |

^a^Number of samples analyzed

^b^Number of metabolites detected

^c^Incidence of contamination expressed in percentage

^d^Values denote peak areas (no quantitative standard available)

**Table S4** Distribution and occurrence of additional microbial metabolites in 28 other ingredients/supplements for poultry feed in Nigeria.

| Metabolites | Bone (*n^a^*= 9; *n^b^*= 59) | | | | Fish meal (*n^a^*= 5; *n^b^*= 25) | | | | | | Palm kernel (*n^a^*= 3; *n^b^*= 68) | | | | Soybean (*n^a^*= 11; *n^b^*= 52) | | | |  |
| --- | --- | --- | --- | --- | --- | --- | --- | --- | --- | --- | --- | --- | --- | --- | --- | --- | --- | --- | --- |
|  | %^c^ | Concentration (µg/kg) | | | |  | | Concentration (µg/kg) | | |  | Concentration (µg/kg) | | |  | Concentration (µg/kg) | | |  |
|  |  | Min | Max | Mean | %^c^ | | Min | | Max | Mean | %^c^ | Min | Max | Mean | %^c^ | Min | Max | Mean | |
| 3-Nitropropionic acid | 11.1 | 24 | 24 | 0.0 | 80 | | 34.0 | | 89 | 49 | 100 | 15 | 32 | 25 | 72.7 | 7.4 | 43 | 15 | |
| Agroclavine | 11.1 | 0.3 | 0.3 | 0.0 | 0.0 | | 0.0 | | 0.0 | 0.0 | 33.3 | 0.5 | 0.5 | 0.0 | 18.2 | 3.2 | 4.2 | 3.7 | |
| Andrastin A | 0.0 | 0.0 | 0.0 | 0.0 | 20 | | 7,600^d^ | | 7,600^d^ | 0.0 | 66.7 | 13,400^d^ | 18,400^d^ | 15,867 | 9.1 | 2,120^d^ | 2,120^d^ | 0.0 | |
| Asperglaucide | 100 | 1.5 | 156 | 36 | 100 | | 17 | | 3,000 | 1,688 | 100 | 173 | 3,760 | 1,382 | 100 | 3.0 | 1,890 | 431 | |
| Aurofusarin | 11.1 | 34 | 34 | 0.0 | 0.0 | | 0.0 | | 0.0 | 0.0 | 0.0 | 0.0 | 0.0 | 0.0 | 0.0 | 0.0 | 0.0 | 0.0 | |
| Averantin | 0.0 | 0.0 | 0.0 | 0.0 | 0.0 | | 0.0 | | 0.0 | 0.0 | 33.3 | 0.2 | 0.2 | 0.0 | 0.0 | 0.0 | 0.0 | 0.0 | |
| Averufanin | 11.1 | 0.7 | 0.7 | 0.0 | 0.0 | | 0.0 | | 0.0 | 0.0 | 33.3 | 0.4 | 0.4 | 0.0 | 9.1 | 0.2 | 0.2 | 0.0 | |
| Averufin | 11.1 | 9.4 | 9.4 | 0.0 | 60 | | 0.9 | | 1.7 | 1.3 | 100 | 6.1 | 10.0 | 7.6 | 54.5 | 0.9 | 15 | 5.4 | |
| Bikav qual | 11.1 | 12 | 12 | 0.0 | 20 | | 90 | | 90 | 0.0 | 33.3 | 19 | 19 | 0.0 | 9.1 | 13 | 13 | 0.0 | |
| Bis(methylthio)gliotoxin | 0.0 | 0.0 | 0.0 | 0.0 | 0.0 | | 0.0 | | 0.0 | 0.0 | 100 | 35 | 91 | 70 | 0.0 | 0.0 | 0.0 | 0.0 | |
| Brevianamid F | 77.8 | 8.4 | 196 | 46 | 100 | | 1,200 | | 3,630 | 2,984 | 100 | 128 | 243 | 184 | 100 | 15 | 1,030 | 182 | |
| Butyrolactone I | 0.0 | 0.0 | 0.0 | 0.0 | 0.0 | | 0.0 | | 0.0 | 0.0 | 33.3 | 588 | 588 | 0.0 | 0.0 | 0.0 | 0.0 | 0.0 | |
| Chanoclavin | 0.0 | 0.0 | 0.0 | 0.0 | 0.0 | | 0.0 | | 0.0 | 0.0 | 100 | 0.5 | 2.7 | 1.3 | 0.0 | 0.0 | 0.0 | 0.0 | |
| Chloramphenicol | 22.2 | 0.2 | 0.7 | 0.4 | 60 | | 0.3 | | 0.7 | 0.5 | 100 | 0.4 | 189 | 63 | 0.0 | 0.0 | 0.0 | 0.0 | |
| Citreoviridin | 0.0 | 0.0 | 0.0 | 0.0 | 0.0 | | 0.0 | | 0.0 | 0.0 | 33.3 | 94 | 94 | 0.0 | 0.0 | 0.0 | 0.0 | 0.0 | |
| Curvularin | 11.1 | 14.1 | 14.1 | 0.0 | 0.0 | | 0.0 | | 0.0 | 0.0 | 33.3 | 28 | 28 | 0.0 | 27.3 | 3.2 | 13.7 | 8.2 | |
| cyclo(L-Pro-L-Tyr) | 100 | 1.8 | 1,360 | 213 | 100 | | 2,560 | | 17,100 | 7,247 | 100 | 1,110 | 2,160 | 1,674 | 100 | 30 | 8,350 | 1,018 | |

**Table S4** Continued

| Metabolites | Bone (*n^a^*= 9; *n^b^*= 59) | | | | Fish meal (*n^a^*= 5; *n^b^*= 25) | | | | | | Palm kernel (*n^a^*= 3; *n^b^*= 68) | | | | Soybean (*n^a^*= 11; *n^b^*= 52) | | | |  |
| --- | --- | --- | --- | --- | --- | --- | --- | --- | --- | --- | --- | --- | --- | --- | --- | --- | --- | --- | --- |
|  | %^c^ | Concentration (µg/kg) | | | |  | | Concentration (µg/kg) | | |  | Concentration (µg/kg) | | |  | Concentration (µg/kg) | | |  |
|  |  | Min | Max | Mean | %^c^ | | Min | | Max | Mean | %^c^ | Min | Max | Mean | %^c^ | Min | Max | Mean | |
| Cytochalasin H | 0.0 | 0.0 | 0.0 | 0.0 | 0.0 | | 0.0 | | 0.0 | 0.0 | 0.0 | 0.0 | 0.0 | 0.0 | 45.5 | 32 | 172 | 101 | |
| Cytochalasin J | 11.1 | 11 | 11 | 0.0 | 0.0 | | 0.0 | | 0.0 | 0.0 | 33.3 | 9.3 | 9.3 | 0.0 | 72.7 | 4.4 | 401 | 116 | |
| Elymoclavine | 0.0 | 0.0 | 0.0 | 0.0 | 0.0 | | 0.0 | | 0.0 | 0.0 | 0.0 | 0.0 | 0.0 | 0.0 | 18.2 | 2.6 | 5.0 | 3.8 | |
| Emodin | 66.7 | 0.0 | 2.8 | 0.6 | 80 | | 0.5 | | 1.0 | 0.8 | 100 | 54 | 93 | 70 | 63.6 | 0.1 | 0.6 | 0.3 | |
| Enniatin A | 11.1 | 0.4 | 0.4 | 0.0 | 0.0 | | 0.0 | | 0.0 | 0.0 | 0.0 | 0.0 | 0.0 | 0.0 | 9.1 | 0.1 | 0.1 | 0.0 | |
| Enniatin A1 | 11.1 | 3.6 | 3.6 | 0.0 | 0.0 | | 0.0 | | 0.0 | 0.0 | 33.3 | 0.1 | 0.1 | 0.0 | 18.2 | 0.1 | 0.7 | 0.4 | |
| Enniatin B | 22.2 | 0.1 | 4.8 | 2.5 | 60 | | 0.0 | | 0.2 | 0.1 | 66.7 | 0.1 | 0.1 | 0.1 | 18.2 | 0.1 | 1.1 | 0.6 | |
| Enniatin B1 | 22.2 | 0.0 | 8.6 | 4.3 | 40 | | 0.0 | | 0.4 | 0.2 | 33.3 | 0.2 | 0.2 | 0.0 | 27.3 | 0.1 | 2.3 | 0.9 | |
| Enniatin B2 | 11.1 | 0.3 | 0.3 | 0.0 | 0.0 | | 0.0 | | 0.0 | 0.0 | 0.0 | 0.0 | 0.0 | 0.0 | 0.0 | 0.0 | 0.0 | 0.0 | |
| Equisetin | 77.8 | 0.1 | 1.0 | 0.3 | 60 | | 0.2 | | 0.3 | 0.2 | 100 | 0.3 | 1.0 | 0.6 | 90.9 | 0.2 | 10 | 4.4 | |
| Equisetin qual | 0.0 | 0.0 | 0.0 | 0.0 | 0.0 | | 0.0 | | 0.0 | 0.0 | 33.3 | 0.5 | 0.5 | 0.0 | 18.2 | 0.1 | 2.1 | 1.1 | |
| Ergocornine | 0.0 | 0.0 | 0.0 | 0.0 | 0.0 | | 0.0 | | 0.0 | 0.0 | 0.0 | 0.0 | 0.0 | 0.0 | 18.2 | 0.8 | 2.8 | 1.8 | |
| Ergocorninin | 11.1 | 0.3 | 0.3 | 0.0 | 0.0 | | 0.0 | | 0.0 | 0.0 | 0.0 | 0.0 | 0.0 | 0.0 | 0.0 | 0.0 | 0.0 | 0.0 | |
| Ergocristine | 11.1 | 23 | 23 | 0.0 | 0.0 | | 0.0 | | 0.0 | 0.0 | 0.0 | 0.0 | 0.0 | 0.0 | 0.0 | 0.0 | 0.0 | 0.0 | |
| Ergocristinine | 11.1 | 6.2 | 6.2 | 0.0 | 0.0 | | 0.0 | | 0.0 | 0.0 | 0.0 | 0.0 | 0.0 | 0.0 | 9.1 | 0.2 | 0.2 | 0.0 | |
| Ergocryptinine | 11.1 | 0.6 | 0.6 | 0.0 | 0.0 | | 0.0 | | 0.0 | 0.0 | 0.0 | 0.0 | 0.0 | 0.0 | 0.0 | 0.0 | 0.0 | 0.0 | |
| Ergometrin | 11.1 | 0.1 | 0.1 | 0.0 | 0.0 | | 0.0 | | 0.0 | 0.0 | 0.0 | 0.0 | 0.0 | 0.0 | 0.0 | 0.0 | 0.0 | 0.0 | |

**Table S4** Continued

| Metabolites | Bone (*n^a^*= 9; *n^b^*= 59) | | | | Fish meal (*n^a^*= 5; *n^b^*= 25) | | | | | | Palm kernel (*n^a^*= 3; *n^b^*= 68) | | | | | | | Soybean (*n^a^*= 11; *n^b^*= 52) | | | | | | | |  |
| --- | --- | --- | --- | --- | --- | --- | --- | --- | --- | --- | --- | --- | --- | --- | --- | --- | --- | --- | --- | --- | --- | --- | --- | --- | --- | --- |
|  | %^c^ | Concentration (µg/kg) | | |  | | Concentration (µg/kg) | | | |  | | Concentration (µg/kg) | | | | |  | | Concentration (µg/kg) | | | | | |  |
|  |  | Min | Max | Mean | %^c^ | | Min | Max | Mean | | %^c^ | | Min | | Max | Mean | | %^c^ | | Min | | | Max | Mean | | |
| Ergosin | 11.1 | 21 | 21 | 0.0 | 0.0 | | 0.0 | 0.0 | 0.0 | | 0.0 | | 0.0 | | 0.0 | 0.0 | | 0.0 | | 0.0 | | | 0.0 | 0.0 | | |
| Ergosinin | 11.1 | 3.7 | 3.7 | 0.0 | 0.0 | | 0.0 | 0.0 | 0.0 | | 0.0 | | 0.0 | | 0.0 | 0.0 | | 0.0 | | 0.0 | | | 0.0 | 0.0 | | |
| Ergotamine | 11.1 | 21 | 21 | 0.0 | 0.0 | | 0.0 | 0.0 | 0.0 | | 0.0 | | 0.0 | | 0.0 | 0.0 | | 0.0 | | 0.0 | | | 0.0 | 0.0 | | |
| Ergotaminine | 11.1 | 2.2 | 2.2 | 0.0 | 0.0 | | 0.0 | 0.0 | 0.0 | | 0.0 | | 0.0 | | 0.0 | 0.0 | | 0.0 | | 0.0 | | | 0.0 | 0.0 | | |
| Festuclavine | 0.0 | 0.0 | 0.0 | 0.0 | 0.0 | | 0.0 | 0.0 | 0.0 | | 66.7 | | 1.8 | | 14 | 8.1 | | 0.0 | | 0.0 | | | 0.0 | 0.0 | | |
| Fumiquinazolin A | 11.1 | 3,130^d^ | 3,130^d^ | 0.0 | 0.0 | | 0.0 | 0.0 | 0.0 | | 33.3 | | 33,600^d^ | | 33,600^d^ | 0.0 | | 0.0 | | 0.0 | | | 0.0 | 0.0 | | |
| Fumiquinazolin D | 0.0 | 0.0 | 0.0 | 0.0 | 0.0 | | 0.0 | 0.0 | 0.0 | | 33.3 | | 31,900 | | 31,900 | 0.0 | | 0.0 | | 0.0 | | | 0.0 | 0.0 | | |
| Fumitremorgin C | 0.0 | 0.0 | 0.0 | 0.0 | 0.0 | | 0.0 | 0.0 | 0.0 | | 33.3 | | 12 | | 12 | 0.0 | | 0.0 | | 0.0 | | | 0.0 | 0.0 | | |
| Fusaric acid | 0.0 | 0.0 | 0.0 | 0.0 | 0.0 | | 0.0 | 0.0 | 0.0 | | 0.0 | | 0.0 | | 0.0 | 0.0 | | 18.2 | | 296 | | | 332 | 314 | | |
| Fusarinolic acid | 0.0 | 0.0 | 0.0 | 0.0 | 0.0 | | 0.0 | 0.0 | 0.0 | | 0.0 | | 0.0 | | 0.0 | 0.0 | | 9.1 | | 164 | | | 164 | 0.0 | | |
| Helvolic acid | 0.0 | 0.0 | 0.0 | 0.0 | 0.0 | | 0.0 | 0.0 | 0.0 | | 33.3 | | 53 | | 53 | 0.0 | | 0.0 | | 0.0 | | | 0.0 | 0.0 | | |
| Kojic acid | 11.1 | 237 | 237 | 0.0 | 80 | | 182 | 555 | 382 | | 100 | | 172 | | 52,200 | 30,971 | | 81.8 | | 65 | | | 1,990 | 402 | | |
| Linamarin | 11.1 | 54 | 54 | 0.0 | 40 | | 203 | 577 | 390 | | 0.0 | | 0.0 | | 0.0 | 0.0 | | 0.0 | | 0.0 | | | 0.0 | 0.0 | | |
| Lotaustralin | 0.0 | 0.0 | 0.0 | 0.0 | 40 | 23 | | 72 | | 48 | | 0.0 | | 0.0 | 0.0 | | 0.0 | | 9.1 | | 14 | 14 | | 0.0 |  |  |
| Macrosporin | 11.1 | 9.6 | 9.6 | 0.0 | 0.0 | | 0.0 | 0.0 | 0.0 | | 0.0 | | 0.0 | | 0.0 | 0.0 | | 18.2 | | 1.1 | | | 1.4 | 1.3 | | |
| Malformin A | 11.1 | 0.6 | 0.6 | 0.0 | 20 | | 0.6 | 0.6 | 0.6 | | 33.3 | | 0.2 | | 0.2 | 0.2 | | 18.2 | | 0.3 | | | 0.5 | 0.4 | | |
| Malformin C | 0.0 | 0.0 | 0.0 | 0.0 | 0.0 | | 0.0 | 0.0 | 0.0 | | 33.3 | | 0.6 | | 0.6 | 0.6 | | 0.0 | | 0.0 | | | 0.0 | 0.0 | | |

**Table S4** Continued

| Metabolites | Bone (*n^a^*= 9; *n^b^*= 59) | | | | Fish meal (*n^a^*= 5; *n^b^*= 25) | | | | | | Palm kernel (*n^a^*= 3; *n^b^*= 68) | | | | Soybean (*n^a^*= 11; *n^b^*= 52) | | | |
| --- | --- | --- | --- | --- | --- | --- | --- | --- | --- | --- | --- | --- | --- | --- | --- | --- | --- | --- |
|  | %^c^ | Concentration (µg/kg) | | | |  | | Concentration (µg/kg) | | |  | Concentration (µg/kg) | | |  | Concentration (µg/kg) | | |
|  |  | Min | Max | Mean | %^c^ | | Min | | Max | Mean | %^c^ | Min | Max | Mean | %^c^ | Min | Max | Mean |
| Methylsulochrin | 11.1 | 0.2 | 0.2 | 0.0 | 0.0 | | 0.0 | | 0.0 | 0.0 | 100 | 3.5 | 42 | 17 | 0.0 | 0.0 | 0.0 | 0.0 |
| Mevinolin | 0.0 | 0.0 | 0.0 | 0.0 | 0.0 | | 0.0 | | 0.0 | 0.0 | 33.3 | 11 | 11 | 0.0 | 0.0 | 0.0 | 0.0 | 0.0 |
| Monactin | 33.3 | 0.0 | 0.1 | 0.1 | 20 | | 0.6 | | 0.6 | 0.0 | 0.0 | 0.0 | 0.0 | 0.0 | 0.0 | 0.0 | 0.0 | 0.0 |
| Monocerin | 11.1 | 24 | 24 | 0.0 | 20 | | 7.6 | | 7.6 | 0.0 | 33.3 | 1.0 | 1.0 | 0.0 | 18.2 | 2.0 | 2.1 | 2.1 |
| Nidurufin | 11.1 | 0.6 | 0.6 | 0.0 | 0.0 | | 0.0 | | 0.0 | 0.0 | 33.3 | 0.2 | 0.2 | 0.0 | 0.0 | 0.0 | 0.0 | 0.0 |
| Nonactin | 0.0 | 0.0 | 0.0 | 0.0 | 20 | | 0.5 | | 0.5 | 0.0 | 0.0 | 0.0 | 0.0 | 0.0 | 0.0 | 0.0 | 0.0 | 0.0 |
| Norsolorinic acid | 11.1 | 0.9 | 0.9 | 0.0 | 60 | | 0.2 | | 1.2 | 0.6 | 100 | 1.4 | 3.4 | 2.1 | 9.1 | 0.3 | 0.3 | 0.3 |
| O-MethylSTER | 11.1 | 0.9 | 0.9 | 0.0 | 0.0 | | 0.0 | | 0.0 | 0.0 | 33.3 | 5.5 | 5.5 | 0.0 | 0.0 | 0.0 | 0.0 | 0.0 |
| Oxaline | 0.0 | 0.0 | 0.0 | 0.0 | 0.0 | | 0.0 | | 0.0 | 0.0 | 33.3 | 1.3 | 1.3 | 0.0 | 0.0 | 0.0 | 0.0 | 0.0 |
| Penicillide | 0.0 | 0.0 | 0.0 | 0.0 | 0.0 | | 0.0 | | 0.0 | 0.0 | 33.3 | 7.5 | 7.5 | 0.0 | 0.0 | 0.0 | 0.0 | 0.0 |
| Pseurotin A | 0.0 | 0.0 | 0.0 | 0.0 | 0.0 | | 0.0 | | 0.0 | 0.0 | 33.3 | 6.1 | 6.1 | 0.0 | 0.0 | 0.0 | 0.0 | 0.0 |
| Pyripyropene A | 0.0 | 0.0 | 0.0 | 0.0 | 0.0 | | 0.0 | | 0.0 | 0.0 | 33.3 | 14 | 14 | 0.0 | 0.0 | 0.0 | 0.0 | 0.0 |
| Quinocitrinine A | 44.4 | 1.2 | 5.0 | 3.1 | 60 | | 5.2 | | 5.7 | 5.4 | 100 | 16 | 49 | 34 | 36.4 | 7.4 | 12 | 8.9 |
| Rugulosin | 0.0 | 0.0 | 0.0 | 0.0 | 0.0 | | 0.0 | | 0.0 | 0.0 | 33.3 | 39 | 39 | 0.0 | 0.0 | 0.0 | 0.0 | 0.0 |
| Rugulusovin | 22.2 | 91 | 100 | 95 | 100 | | 168 | | 486 | 258 | 100 | 18 | 20 | 23 | 81.8 | 3.2 | 89 | 17 |
| Secalonic acid D | 11.1 | 24 | 24 | 0.0 | 0.0 | | 0.0 | | 0.0 | 0.0 | 0.0 | 0.0 | 0.0 | 0.0 | 0.0 | 0.0 | 0.0 | 0.0 |
| Siccanol | 11.1 | 4,270^d^ | 4,270^d^ | 0.0 | 0.0 | | 0.0 | | 0.0 | 0.0 | 33.3 | 3,160^d^ | 3,160^d^ | 0.0 | 0.0 | 0.0 | 0.0 | 0.0 |
| Skyrin | 11.1 | 0.6 | 0.6 | 0.0 | 0.0 | | 0.0 | | 0.0 | 0.0 | 100 | 10 | 39 | 25 | 0.0 | 0.0 | 0.0 | 0.0 |

**Table S4** Continued

| Metabolites | Bone (*n^a^*= 9; *n^b^*= 59) | | | | Fish meal (*n^a^*= 5; *n^b^*= 25) | | | | Palm kernel (*n^a^*= 3; *n^b^*= 68) | | | | Soybean (*n^a^*= 11; *n^b^*= 52) | | | |
| --- | --- | --- | --- | --- | --- | --- | --- | --- | --- | --- | --- | --- | --- | --- | --- | --- |
|  | %^c^ | Concentration (µg/kg) | | |  | Concentration (µg/kg) | | |  | Concentration (µg/kg) | | |  | Concentration (µg/kg) | | |
|  |  | Min | Max | Mean | %^c^ | Min | Max | Mean | %^c^ | Min | Max | Mean | %^c^ | Min | Max | Mean |
| Sterigmatocystin (STER) | 22.2 | 0.7 | 1.8 | 1.3 | 20 | 4.1 | 4.1 | 0.0 | 33.3 | 3.6 | 3.6 | 0.0 | 45.5 | 0.4 | 1.0 | 0.8 |
| Tentoxin | 11.1 | 3.5 | 3.5 | 0.0 | 0.0 | 0.0 | 0.0 | 0.0 | 0.0 | 0.0 | 0.0 | 0.0 | 0.0 | 0.0 | 0.0 | 0.0 |
| Territrem B | 0.0 | 0.0 | 0.0 | 0.0 | 0.0 | 0.0 | 0.0 | 0.0 | 33.3 | 9.7 | 9.7 | 0.0 | 0.0 | 0.0 | 0.0 | 0.0 |
| Tryptophol | 11.1 | 702 | 702 | 0.0 | 0.0 | 0.0 | 0.0 | 0.0 | 0.0 | 0.0 | 0.0 | 0.0 | 90.9 | 140 | 1,160 | 668 |
| Versicolorin A | 11.1 | 0.1 | 0.1 | 0.0 | 0.0 | 0.0 | 0.0 | 0.0 | 100 | 0.4 | 1.8 | 1.0 | 9.1 | 0.4 | 0.4 | 0.0 |
| Versicolorin C | 11.1 | 1.5 | 1.5 | 0.0 | 0.0 | 0.0 | 0.0 | 0.0 | 100 | 2.0 | 4.4 | 3.1 | 63.6 | 0.2 | 5.0 | 1.0 |

^a^Number of samples analyzed

^b^Number of metabolites detected

^c^Incidence of contamination expressed in percentage

^d^Values denote peak areas (no quantitative standard available)
